# Metformin induces tolerogenicity of dendritic cells by promoting metabolic reprogramming

**DOI:** 10.1007/s00018-023-04932-3

**Published:** 2023-09-09

**Authors:** Xianmei Liu, Peng Yu, Yujun Xu, Yun Wang, Jin Chen, Fuzhou Tang, Zuquan Hu, Jing Zhou, Lina Liu, Wei Qiu, Yuannong Ye, Yi Jia, Weijuan Yao, Jinhua Long, Zhu Zeng

**Affiliations:** 1 School of Basic Medical Sciences/School of Biology and Engineering, Guizhou Medical University, Guiyang, 550025 People’s Republic of China; 2 Key Laboratory of Infectious Immunity and Antibody Engineering in Guizhou Province/Engineering Center of Cellular Immunotherapy in Guizhou Province, Guiyang, 550025 People’s Republic of China; 3 Department of Interventional Radiology, Affiliated Hospital of Guizhou Medical University, Guiyang, 550004 People’s Republic of China; 4 Department of Head & Neck, Affiliated Tumor Hospital of Guizhou Medical University, Guiyang, 550004 People’s Republic of China; 5 Hemorheology Center, Department of Physiology and Pathophysiology, School of Basic Medical Sciences, Peking University Health Science Center, Beijing, 100191 People’s Republic of China; 6 Key Laboratory of Endemic and Ethnic Diseases, Ministry of Education, Guizhou Medical University, Guiyang, 550004 Guizhou People’s Republic of China; 7 State Key Laboratory of Functions & Applications of Medicinal Plants, Guizhou Medical University, Guiyang, 550004 People’s Republic of China

**Keywords:** Dendritic cells, Tolerant dendritic cells, Metformin, Immune tolerance, Immune metabolism, Autoimmune diseases

## Abstract

Dendritic cells (DCs) can mediate immune responses or immune tolerance depending on their immunophenotype and functional status. Remodeling of DCs’ immune functions can develop proper therapeutic regimens for different immune-mediated diseases. In the immunopathology of autoimmune diseases (ADs), activated DCs notably promote effector T-cell polarization and exacerbate the disease. Recent evidence indicates that metformin can attenuate the clinical symptoms of ADs due to its anti-inflammatory properties. Whether and how the therapeutic effects of metformin on ADs are associated with DCs remain unknown. In this study, metformin was added to a culture system of LPS-induced DC maturation. The results revealed that metformin shifted DC into a tolerant phenotype, resulting in reduced surface expression of MHC-II, costimulatory molecules and CCR7, decreased levels of proinflammatory cytokines (TNF-α and IFN-γ), increased level of IL-10, upregulated immunomodulatory molecules (ICOSL and PD-L) and an enhanced capacity to promote regulatory T-cell (T_reg_) differentiation. Further results demonstrated that the anti-inflammatory effects of metformin in vivo were closely related to remodeling the immunophenotype of DCs. Mechanistically, metformin could mediate the metabolic reprogramming of DCs through FoxO3a signaling pathways, including disturbing the balance of fatty acid synthesis (FAS) and fatty acid oxidation (FAO), increasing glycolysis but inhibiting the tricarboxylic acid cycle (TAC) and pentose phosphate pathway (PPP), which resulted in the accumulation of fatty acids (FAs) and lactic acid, as well as low anabolism in DCs. Our findings indicated that metformin could induce tolerance in DCs by reprogramming their metabolic patterns and play anti-inflammatory roles in vitro and in vivo.

**Supplementary Information:**

The online version contains supplementary material available at 10.1007/s00018-023-04932-3.

## Introduction

Dendritic cells (DCs), as the most potent professional antigen presenting cells, are the core element of the immune system and control the immune response by priming immune responses through the activation of naïve T cells or mediating immune tolerance under homeostatic conditions [1, 2]. Immature DCs (imDCs) are mainly responsible for antigen capture and processing, have a low ability to stimulate T-cell activation and usually mediate immune tolerance by inducing T-cell anergy or regulatory T cells (T_regs_) differentiation [2, 3]. After encountering antigens while maturing, DCs can secrete more proinflammatory cytokines (IL-12, IL-6, IL-1β, TNF-α, etc.), upregulate the expression of MHC-II, costimulatory molecules (CD80, CD86, and CD40) and CCR7, which enable these cells to rapidly migrate to lymphatic tissues and exhibit strong antigen presentation to drive naïve T-cell polarization into proinflammatory Th_1_, Th_2_, Th_17_ cells or cytotoxic T lymphocytes (CTLs) [4–6]. Once the maturation process is disturbed or retarded, DCs express insufficient immunophenotypic molecules on their cell surface, change their cytokine profile (increased secretion of IL-10, TGF-β, etc.) [7, 8] and upregulate the expression levels of immunoregulatory molecules such as programmed cell death ligand (PDL)-1, heme oxygenase-1 (HO-1), inducible T-cell costimulatory ligand (ICOSL) and indoleamine 2,3-dioxygenase (IDO) [9–11], and the resulting DCs are semimature DCs or tolerant DCs (tolDCs) [12], which are involved in promoting T_reg_ differentiation and suppressing effector T-cell polarization, leading to immune tolerance. As a result, the functional status of DCs is closely associated with the polarization of T cells, which determines the immune response type. Altering DC immune functions could remodel the immune response in the body. Autoimmune diseases (ADs) are caused by disruptions in the immune system, including hyperactivation of adaptive immune responses and chronic inflammation [13, 14]. Increased hyperactive effector T cells (Th_1_, Th_2_ and Th_17_, etc.) and decreases in T_reg_ in organs and tissues are the main reasons that the immune system escapes immune checkpoints controlled by the tolerogenic arm of the immune system [14–16]. In ADs, DCs are mostly mature with high expression of MHC-II and costimulatory molecules and secrete sufficient amounts of IL-12, TNF-α and IL-1β, exerting significant proinflammatory activity and exacerbating the disequilibrium of effector T cells/T_regs_, which promotes disease occurrence and development [17–19]. Therefore, it is inferred that targeting the functional status of DC could explore new regimens for treating ADs, such as reprogramming DC into a tolerant phenotype by medicine or bioengineering technology.

Recently, it was found that metformin, a first-line drug for type 2 diabetes, can attenuate the clinical symptoms of ADs due to its anti-inflammatory properties [20, 21]. Studies have indicated that metformin can significantly inhibit the immune pathological process of ADs by rebalancing the ratio of hyperactive effector T cells/T_regs_ by reducing the proportion of Th_1_ and Th_17_ cells and increasing the number of T_regs_ in multiple animal models of ADs [22, 23]. Metformin could also dramatically lower the levels of proinflammatory cytokines such as IL-12, TNF-α, IL-6 and IL-1β and ameliorate the severity of ADs in animal models [24, 25]. Monta et al. reported that genes involved in the immune response and inflammatory regulation were downregulated in healthy subjects by oral metformin administration, suggesting that metformin possesses anti-inflammatory properties [26]. This evidence suggests that metformin may be an effective candidate drug for the treatment of ADs. However, until now, the underlying mechanisms of the immunomodulatory effects of metformin, especially its role in reprogramming the polarization of T cells into different subpopulations, remain largely unclear. It is well known that, after receiving antigen stimulation, naïve T cells can differentiate into different T-cell subsets, and this process is closely related to the immune functions of DCs [4, 27]. Thus, it is reasonable to infer that the immunomodulatory effects of metformin may mediated by modulating the immunophenotype and immune functions of DCs.

Immunometabolism is the crosstalk between the immune system and metabolism [28–30]. Diverse immune cell subsets and cells in different differentiation stages have unique metabolic patterns to meet energy and biosynthesis demands and adapt their immune functions. For example, naïve T cells rarely participate in the immune response using oxidative phosphorylation (OXPHOS) to sustain their functions [31, 32]; effector T cells (Th_1_, Th_2_ and Th_17_) with enhanced aerobic glycolysis and OXPHOS can amplify the proinflammatory response [33]; and T_regs_ guide immune tolerance mainly by using fatty acid oxidation (FAO) and catabolism to meet their energy demands [34]. During the development and maturation of DCs, the transformation of the metabolic mode (OXPHOS and catabolism for imDCs and aerobic glycolysis and anabolism for mature DCs (mDCs)) ensures that DCs obtain sufficient energy to maintain their various biosynthesis and immunological behaviors [35, 36]. Thus, metabolism is a key regulator that guides the fate of immune cells and shapes their immune functions. Pharmacologically, metformin can regulate metabolic patterns to tailor cell biological behaviors and functions [37–40]. Based on these results, metformin may rewire the immunophenotype and immune functions of DCs by regulating their metabolic patterns, further shaping the polarization of naïve T cells and mediating the resulting immune response.

In the present study, the results showed that metformin could turn LPS-induced DCs into tolDCs, promoting the differentiation and proliferation of T_regs_ and mediating immune tolerance in vitro and in vivo. Mechanistically, metformin disturbed the balance of fatty acid synthesis (FAS) and FAO, increased the utilization of glucose by DCs, increased glycolysis, and inhibited tricarboxylic acid cycle (TAC) and pentose phosphate pathway (PPP) through FoxO3a signaling pathways, leading to the accumulation of fatty acids (FAs) and lactic acid, as well as low anabolism in DCs. In light of these results, metformin could be a preferable candidate for remodeling the immunophenotype and immune functions of DC and reprogramming the immune response outcome of the body. Our findings are benefit for further understanding the immunoregulatory mechanism of metformin and provide a theoretical basis for the use of metformin in immune-related diseases.

## Materials and methods

### Reagents and antibodies

Recombinant murine granulocyte–macrophage colony stimulating factor (rmGM-CSF), recombinant murine interleukin-4 (rhIL-4) and murine MIP-3β (CCL19) were purchased from Peprotech (Rocky Hill, NJ, USA). LPS and FITC-conjugated dextran (molecular weight: 43 kDa) were purchased from Sigma‒Aldrich (St. Louis, MO, USA). Metformin was purchased from Medchem Express (New Jersey, USA). FITC-conjugated anti-mouse CD80/CD86/CD4, PE-conjugated anti-mouse CD40/MHC-II/CCR7/Foxp3/PD-L1, and APC-conjugated anti-mouse CD11c/CD25 antibodies were purchased from Thermo Fisher Scientific (Waltham, MA, USA). Mouse IL-12p40, IL-6, TNF-α, IFN-γ, IL-10 and TGF-β enzyme-linked immunosorbent assay (ELISA) kits were obtained from 4A Biotech Co., Ltd (Beijing, China). Antibodies against phosphorylated forkhead box-O3a (pFoxO3a) (s253) and Foxo3a were obtained from Huabio Co., Ltd. (Hangzhou, China). Antibodies against GAPDH, TRITC phalloidin, 40,6-diamidino-2-phenylindole (DAPI) and Cell Counting Kit 8 (CCK-8) were obtained from Solarbio (Beijing, China). A mouse naïve CD4^+^ T-cell isolation kit was purchased from Miltenyi Biotec (Cologne, Germany). Hematoxylin and eosin (H&E) staining solution was obtained from Thermo Fisher Scientific (Waltham, MA, USA).

### Animals and ethics

The male animals (C57BL/6 mice) used in the study, cultured bone marrow-derived DCs (BMDCs) and the mouse model of inflammatory bowel disease (IBD), were handled in accordance with the requirements of the guidelines for animal experiments, which were approved by the Animal Ethics Committee of Guizhou Medical University. The number of the animal experimental ethical inspection form is 1,900,272.

### BMDC culture and treatment with metformin

BMDCs were prepared as described in our previous study [8]. Briefly, bone marrow cells were flushed from freshly dissected femurs and tibias of male C57BL/6 mouse (7 weeks old). Lysis buffer was used to remove the red blood cells. The remaining cells were cultured in Roswell Park Memorial Institute 1640 (RPMI 1640) complete medium with 10% fetal bovine serum, 1% penicillin‒streptomycin, 0.1% β-mercaptoethanol, 20 ng/ml rmGM-CSF and 10 ng/ml rmIL-4 for 7 days to obtain imDCs, which were then matured into mDCs by adding 100 ng/ml LPS and incubating for another 24 h.

It is well known that metformin was greatest accumulated in the gastrointestinal tract, particularly the jejunum and ileum, after oral administration [41]. Studies showed that the concentration of metformin in the jejunum was about 10–50 times higher than plasma concentrations for mice [41], and 30–300 times for human [42], which indicated that the concentration of metformin in the jejunum of mice can be as high as 0.69 ~ 3.47 mM (50 mg/kg, 0.5 h after oral) and 0.24 ~ 7.2 mM of human (850 mg, 3 h after oral). Since animal experiments in the present study focused on the effect of metformin in the treatment of IBD, we selected millimolar (mM) levels of metformin concentration as the treatment concentration in in vitro experiments and further determined its treatment concentration and time according to the cell viability assay by CCK-8 kit. Therefore, during the maturation of LPS-induced imDCs, different concentrations of metformin (0, 1, 2, 4, 8 mM) were added to the culture medium to achieve mDCs (LPS + 0 mM metformin) and Met-mDCs (LPS + 1, 2, 4, 8 mM metformin). Met-mDCs specifically refer to cells that were treated with LPS and 4 mM metformin in the following experiments.

### Flow cytometry

To analyze the effects of metformin on the expression levels of DC molecules, imDCs, mDCs and Met-mDCs were labeled with FITC-, PE-, APC-conjugated CD80, CD86, CD40, MHC-II, CCR7 and CD11c Abs. Then, the cells were measured by a FACS Calibur cytometer (BD Biosciences, NJ, USA), and double-positive cells (CD11c -CD80/CD86/CD40/MHC-II/CCR7) were analyzed. In the animal assays, spleen cells and mesenteric lymph node (MLN) cells were incubated for 20 min at room temperature with anti-CD11c, anti-CD80, anti-PD-L1 to detect DCs and anti-CD4, anti-CD25, and anti-Foxp3 antibodies to T_regs_.

### Measurement of cytokines by ELISA

The cell culture supernatants of imDCs, mDCs and Met-mDCs were collected and centrifuged at 1200 rpm for 5 min. The supernatants were transferred into new EP tubes and fully mixed. Then, the concentrations of IL-12p40, TNF-α, IL-6, IFN-γ, IL-10 and TGF-β in the culture medium were detected by ELISA kits according to the manufacturer’s instructions.

### Mixed lymphocyte reaction (MLR) and T_reg_ differentiation

Allogeneic spleen-derived T cells were separated by a nylon wool column (Polysciences, PA, USA) according to the manufacturer’s protocols. Then, imDCs, mDCs and Met-mDCs were separately cocultured with T cells (DCs: T cells = 1:1, 1:10, 1:50 and 1:100) in a 37 °C incubator with 5% CO_2_ for 48 h. Then, the cocultured cell suspensions were collected and fully mixed. Then, 100 μl of these mixtures was seeded in a 96-well plate, and 10 μl of CCK-8 solution was added. After 4 h, T-cell proliferation was quantified by measuring the optical density at 450 nm with a microplate reader (Bio-Rad, Hercules, CA, USA). To analyze T_reg_ differentiation, the cocultured cells in each group (1:10 mix ratio) were labeled with FITC-conjugated CD4, APC-conjugated CD25 and PE-conjugated Foxp3 Abs and then measured by flow cytometry. Triple-positive cells were identified and analyzed by flow cytometry.

### Endocytosis and migration assay

To detect DC endocytosis, 1 × 10^6^ imDCs, mDCs and Met-mDCs were precooled for 30 min and then incubated with FITC-dextran particles (final concentration: 1 mg/ml) at 4 °C or 37 °C for 2 h. After the cells were washed three times with PBS, the mean fluorescence intensities were analyzed by flow cytometry. To compare the migration of mDCs and Met-mDCs, a transwell assay was performed. Briefly, a total of 2 × 10^5^ cells in a 200 μl volume were added to the upper chamber (5-mm pore size), and 600 μl of RPMI 1640 complete medium with 200 ng/ml CCL19 (for chemotaxis) was added to the lower chamber. The transwell device was placed in a 37 °C incubator, and the cells were allowed to migrate for 24 h. Then, the migrated cells were counted under a light microscope.

### Real-time quantitative PCR (RT‒qPCR)

Total RNA was extracted from imDCs, mDCs and Met-mDCs using an ESscience RNA-Quick Purification Kit (YiShan Biotech, Shanghai, China) and reverse-transcribed into cDNA with the 5 × FastKing-RT SuperMix Kit (Tiangen Biotech, Beijing, China) for RT‒qPCR, which was performed on a quantitative real-time amplification system (Bio-Rad, Hercules, CA, USA). Relative RNA expression was calculated by the 2^−ΔΔCt^ method with normalization to GAPDH. The primer sequences are listed in Supplemental Table 1.

### Western blotting

Total protein was extracted from imDCs, mDCs and Met-mDCs using RIPA buffer containing protease (phenylmethylsulfonylfluoride (PMSF)) and phosphatase inhibitors (Solarbio, Beijing, China). The protein concentration was determined by a BCA kit (Servicebio, Beijing, China). Equal amounts of denatured protein samples were separated on a 10% sodium dodecyl sulfate‒polyacrylamide gel electrophoresis (SDS‒PAGE) gel and transferred onto 0.45 μm PVDF membranes (Merck Millipore Ltd., Tullagreen, Carrigtwohill Co. Cork, Ireland). After being blocked with 5% nonfat milk, the membranes were incubated with primary antibodies against GAPDH, pFoxO3a (s253) and Foxo3a overnight at 4 °C and then with HRP-conjugated secondary antibodies for 1 h at room temperature. The signals were visualized using an enhanced chemiluminescence detection kit (Meilunbio, Dalian, China) in an imaging system (Bio-Rad, Hercules, CA, USA). The mean gray value of each band was analyzed by ImageJ software.

### Untargeted metabolic analysis

Approximately 1 × 10^7^ imDCs, mDCs and Met-mDCs (six duplicate samples of each group) were harvested and washed twice with 0.9% NaCl. The cell pellets were then quickly frozen with liquid nitrogen and transported on dry ice to Shanghai Applied Protein Technology Co., Ltd. Untargeted metabolomics profiling of the cells was performed using ultrahigh-performance liquid chromatography (Agilent 1290 Infinity LC) coupled with quadrupole time-of-flight mass spectrometry (UHPLC-QTOF/MS). The number of the untargeted metabolomics profile is P20210200970.

### Extracellular oxygen consumption assay and FAO assay

The extracellular oxygen consumption of DCs was measured by a kit (ab197243, Abcam, Cambridge, UK). An FAO kit (ab217602, Abcam, Cambridge, UK) combined with an extracellular oxygen consumption kit (ab197243) was used to detect the level of FAO in DCs according to the manufacturer’s instructions. Briefly, mDCs and Met-mDCs were seeded in a 96-well plate at a density of 5–6 × 10^5^ cells/well in 150 μl of culture medium, and cell-free wells were used as the blank control or signal control. Then, the corresponding reagents were added to the wells according to the kit protocols. Extracellular O_2_ consumption and FAO levels were measured with Cytation 5 (Bio Tek, Vermont, USA) at 1.5 min intervals for 120 min using the following excitation and emission wavelengths: Ex/Em = 380/650 nm.

### Measurement of glucose consumption and extracellular acidification

To measure glucose consumption, 600 μl of 1 × 10^6^/ml mDCs and Met-mDCs were seeded in 24-well plates for 24 h. Then, the culture supernatant was harvested, and the glucose concentration was measured according to the instructions of the glucose measuring kit (Rsbio, Shanghai, China). The glucose concentration in RPMI 1640 complete medium without cells was used as the initial concentration. Glucose consumption was calculated with the following formula: (initial glucose concentration –measured glucose concentration) × volume (600 μl).

The extracellular acidification of DCs was measured using a glycolysis assay kit (Abcam, Cambridge, UK) according to the manufacturer’s instructions. Briefly, mDCs and Met-mDCs were harvested and washed twice with respiration buffer. Approximately 4.5 × 10^5^ cells in 150 μl of respiration buffer were seeded in a 96-well plate, and cell-free respiration buffer wells with or without glucose oxidase (Solarbio, Beijing, China) was used as the positive control and blank control, respectively. Then, 10 μl of reconstituted glycolysis assay reagent was added to each sample well and positive control well. Ten microliters of respiration buffer were added to the blank control wells. Then, the prepared plate was inserted into Cytation 5 (Bio Tek, Vermont, USA), which had been preheated to 37 °C, and the glycolysis assay signal was measured at 1.5 min intervals for 150 min using the following excitation and emission wavelengths: Ex/Em = 380/615 nm.

### Measurement of lactic acid in DCs and their culture medium

The level of lactic acid in DCs and their culture medium was measured by a lactic acid detection kit (Solarbio, Beijing, China). In brief, 5 × 10^6^ mDCs and Met-mDCs, as well as their culture medium, were collected. According to the manufacturer’s protocols, the extract was added to the DC pellets and fully mixed, and then the cells were disrupted by ultrasonic waves (power, 300 W; disruption, 25 s; interval, 25 s; total, 3 min) in an ice bath to release the lactic acid. Likewise, after the extract was added and fully mixed, the culture medium was centrifuged at 12,000 × *g* for 10 min at 4 °C. Then, the supernatants were collected and placed on ice for analysis. The lactic acid content in each group was measured and calculated according to the kit instructions.

### Animal experiments

C57BL/6 mice were administered 3% (W/V) dextran sulfate sodium (DSS) in drinking water for 7 days to induce colitis. The mice were treated with metformin oral administration or DC-based cell therapy. For metformin oral administration as shown in Fig. 5a: thirty-two male mice (6 weeks old) were randomly divided into four groups as follows: (1) the normal control (NC) group received normal drinking water; (2) the normal control + PBS (NP) group received normal drinking water and were treated daily with PBS by gavage; (3) the DSS + PBS (DP) group received 3% DSS in drinking water and daily PBS treatment by gavage; and (4) the DSS + Metformin (DM) group received 3% DSS in drinking water and daily metformin (300 mg/kg^.^d) treatment by gavage. For DC-based cell therapy as shown in supplemental Fig. 6a: thirty male mice (6 weeks old) were randomly divided into five groups: the non-sick mice (NSM) group, the sick mice + PBS (SM-PBS) group, the sick mice + imDC (SM-imDC) group, the sick mice + mDC (SM-mDC) group and the sick mice + Met-mDC (SM-Met-mDC) group. The NSM group received normal drinking water and without treatment; the latter four received 3% DSS in drinking water for 7 days and treatment with 100 μl PBS or 1 × 10^6^ imDCs/mDCs/Met-mDCs in 100 μl PBS intraperitoneal injection on days 4 and 7, respectively. All mice received a normal diet. Body weight loss, the presence of blood in stool and stool consistency were monitored and recorded daily. The disease activity index was calculated as previously described [43]. The detailed scoring rubric is shown in Supplemental Table 2. On the eighth or tenth day, the blood was collected by eyeball extirpating after the mice were anesthetized; then the mice were executed by cervical dislocation, followed by collection of the colons, MLNs and spleen. The whole blood cells analysis was examined with an automatic hemocyte analyzer (Mindray, Shenzhen, China) within 1 h after blood sampling, then the counts of white blood cell (WBC)/granuocyte/lymphocyte were obtained from the results were further analyzed and compared. The serum level of IL-10 was measured by an ELISA kit (4A Biotech, Beijing, China).

### Histological H&E staining

Colon length was measured immediately after excised, and then the colons were fixed in 4% paraformaldehyde for further histopathological analysis using H&E staining. Briefly, after fixation, the colons were longitudinally embedded in paraffin, sectioned at a thickness of 3–5 μm, and then stained with H&E according to standardized protocols. The slides were observed under an orthographic microscope (Nikon Eclipse E100; Nikon, Tokyo, Japan), and images were captured by an imaging system (Nikon DS-U3; Nikon, Tokyo, Japan).

### Immunofluorescence microscopy

MLNs and spleen were excised and fixed in 4% paraformaldehyde. The tissues were then embedded in paraffin and sectioned at a thickness of 3–5 μm. After dewaxing, dehydration and antigenic reparation, the slices were stained overnight at 4 °C with primary antibodies against CD4, Foxp3, CD80, PD-L1 or CD11c (Servicebio, Wuhan, China) and incubated with HRP-conjugated or FITC-conjugated secondary antibodies (Servicebio, Wuhan, China) for 50 min at room temperature. Cell nuclei were labeled with DAPI for 5 min. The slices were sealed with an anti-fluorescence quenching agent (Servicebio, Wuhan, China) and imaged by fluorescence microscopy (Nikon, Tokyo, Japan).

### Laser scanning confocal microscopy

imDCs, mDCs and Met-mDCs were cultured on poly L-lysine-treated coverslips for 4 h and fixed in 4% paraformaldehyde for 20 min. After being permeabilized with 0.1% Triton X-100 and blocked with 1% bovine serum albumin (BSA), the cells were incubated with pFoxO3a (s253) overnight at 4 °C and labeled with FITC-conjugated secondary antibodies (Abcam, Cambridge, UK) for 1 h at room temperature. Then, the cells were stained with rhodamine phalloidin (Solarbio, Beijing, China) and DAPI in the dark for 20 min and 5 min, respectively. The cells were imaged using a laser scanning confocal microscope (Olympus or Nikon, Tokyo, Japan).

### Statistical analysis

The data are presented as the mean ± standard deviation (SD). Paired or unpaired Student’s t tests were used for comparisons between two groups. One-way ANOVA or two-way ANOVA were used for multiple group comparisons. Prism 8.0 software (GraphPad, CA, USA) was used to analyze all data and draw statistical graphs (*p < 0.05; **p < 0.01; ***p < *0.005*)*.*

## Results

### Metformin slowed the maturation of DCs

**Fig. 1:**
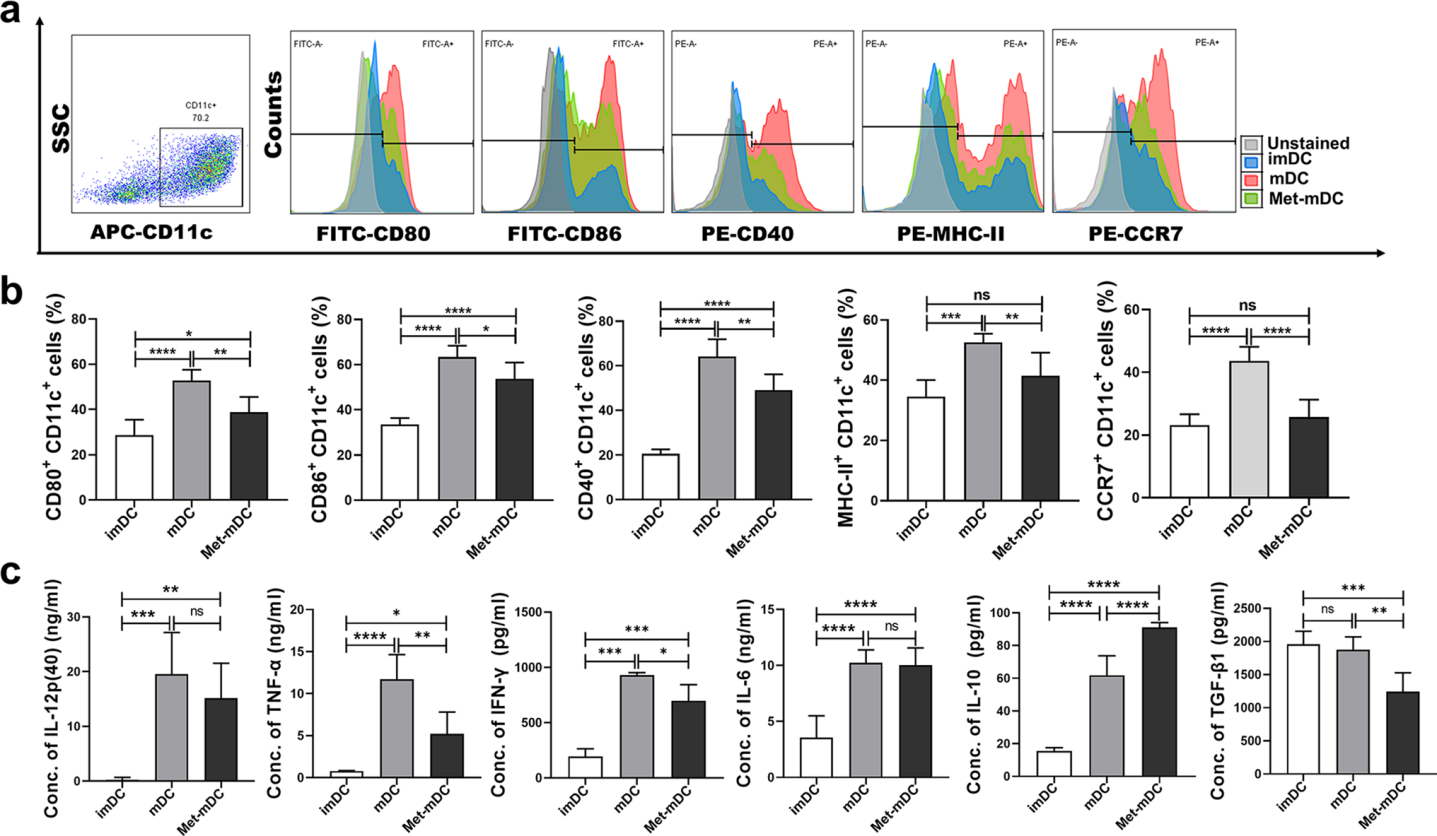
Metformin inhibited the LPS-induced upregulation of immunophenotypic molecules and the secretion of proinflammatory cytokines in DCs while increasing the secretion of IL-10. **a** Flow cytometry was performed to detect the expression of CD80, CD86, CD40 MHC-II and CCR7 in the CD11c^+^ cell population of imDCs, mDCs and Met-mDCs. **b** The corresponding statistical histograms for the data in (A). N = 5–6. **c** The culture medium of imDCs, mDCs and Met-mDCs was collected, and then the concentrations of IL-12p(40), TNF-α, IFN-γ, IL-6, IL-10 and TGF-β were measured by ELISA kits. N = 4–5. One-way ANOVA was used for comparisons among three groups. Mean ± SD *p < 0.05; **p < 0.01; ***p < 0.001; ****p < 0.0001

**Fig. 2:**
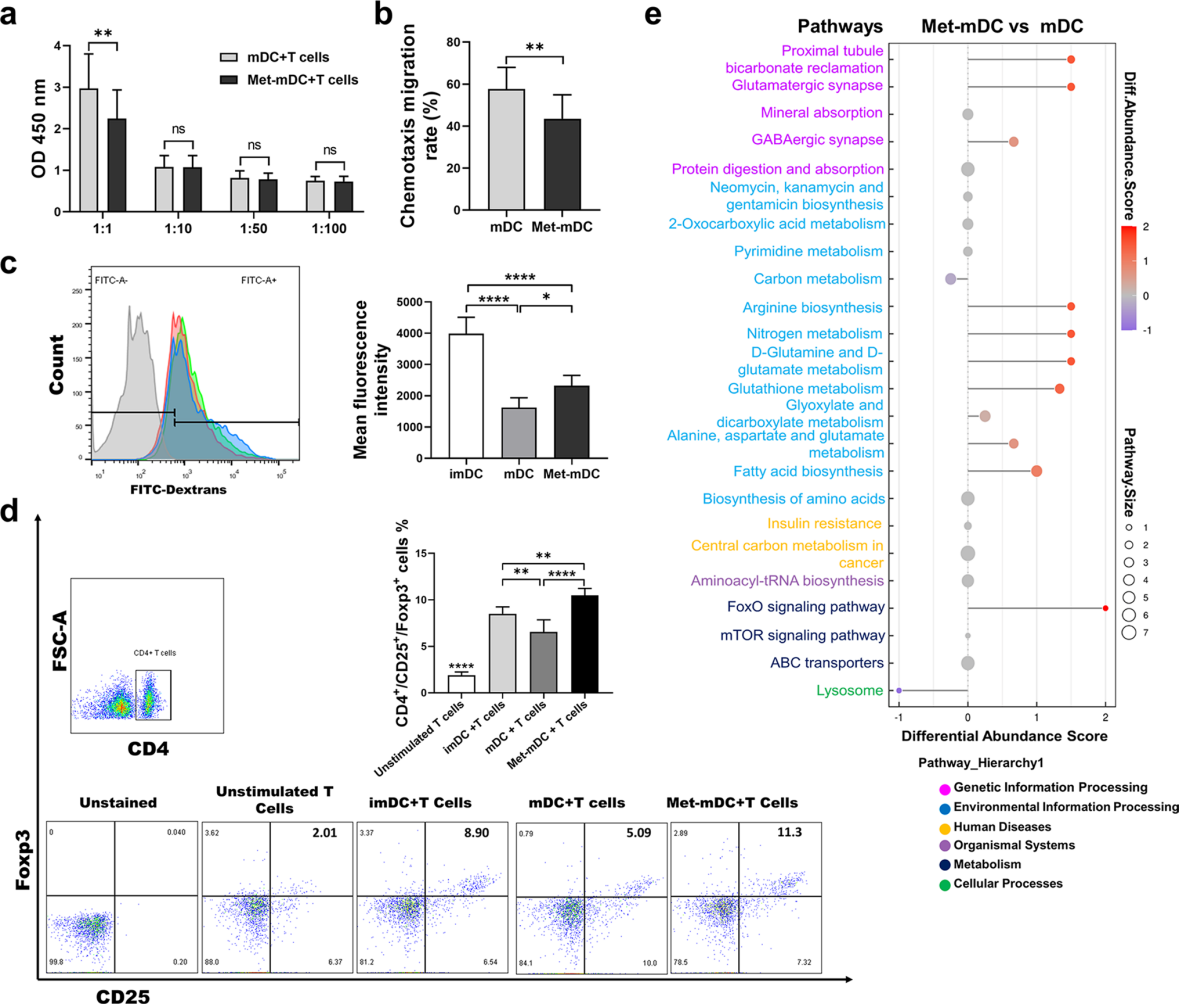
Metformin impaired the T-cell stimulatory capacity of DCs and shifted DCs into a tolerant phenotype. **a** The T-cell stimulatory capacity of mDCs and Met-mDCs was measured by an allogeneic mixed lymphocyte reaction assay with coculture ratios (DCs: T cells) of 1:1, 1:10, 1:50 and 1:100. N = 7. **b** Chemotactic migration of mDCs and Met-mDCs toward CCL19 (200 ng/mL) was examined using a transwell system. N = 5. **c** For endocytosis analysis, imDCs, mDCs and Met-mDCs were incubated with FITC-dextran particles for 2 h, and the mean fluorescence intensities were measured by flow cytometry. N = 5. **d** To analyze T_reg_ differentiation, cocultures of DCs and spleen-derived naïve T cells at a ratio of 1:10 were labeled with FITC-conjugated CD4, APC-conjugated CD25 and PE-conjugated Foxp3 Abs, and triple-positive cells were measured by flow cytometry. N = 6. **e** KEGG pathway enrichment analysis of differential metabolites in the untargeted metabolomics data of Met-mDCs and mDCs. N = 6. Paired Student’s t tests were used for two-group comparisons. One-way ANOVA was used for comparisons among three groups. Mean ± SD *p < 0.05; **p < 0.01; ***p < 0.001

The viability of DCs treated with metformin (Supplemental Fig. 1a) was measured by a CCK-8 kit (Supplemental Fig. 1b) to determine the working concentration and treatment time. As shown in supplemental Fig. 2a, 4 mM metformin significantly inhibited the LPS-induced upregulation of immunophenotypic molecules (CD40, MHC-II and CCR7) in DCs. Therefore, the working concentration of metformin was 4 mM for 24 h in the following experiments. LPS stimulated the maturation process in DCs, resulting in dramatic increased expression of CD80, CD86, CD40, MHC-II and CCR7 and high production of IL-12p(40), TNF-α, IFN-γ, IL-6 and IL-10, and when metformin was added to the culture medium in the presence of LPS, the upregulation of these molecules and cytokines, including TGF-β, was significantly inhibited, and IL-10 was further increased (Fig. 1). The results suggested that the maturation of Met-mDCs was deficient. With the maturation of DCs, their ability to stimulate primary T-cell responses was enhanced, and DCs exhibit strong migration induced by CCR7-CCL19/21, while their endocytic ability was frequently weakened [44, 45]. To further confirm the inhibitory effect of metformin on DC maturation, the immune functions of mDCs and Met-mDCs were investigated. Allogeneic MLR results showed that Met-mDCs induced markedly weaker T-cell activation at a 1:1 mixture ratio of DCs and T cells (Fig. 2a). Met-mDCs also showed obvious reduced chemotaxis (Fig. 2b), which was consistent with the decreased expression level of CCR7 in Met-mDCs (Fig. 1a, b). Then, imDCs, mDCs and Met-mDCs were incubated with FITC-labeled dextran for 2 h, and we found that, compared with imDCs, the endocytic ability of mDCs was weakened, while metformin could rescue this ability when it was administered during LPS-induced DC maturation (Fig. 2c). Overall, these results suggested that metformin could inhibit LPS-induced DC maturation.

### Metformin shifted DCs into a tolerant phenotype

Recently, tolDCs were defined as a type of DCs that lack proinflammatory cytokine production, possess a semimature phenotype and mediate immune tolerance [12, 46]. Further studies showed that tolDCs express low levels of MHC-II, costimulatory molecules and proinflammatory cytokines and high levels of anti-inflammatory cytokines (IL-10, TGF-β, etc.) and immunomodulatory molecules (PD-L1, HO-1, ICOSL, IDO, etc.), leading to naïve T-cell polarization into T_regs_ [9, 47, 48]. Therefore, to further elucidate the effect of metformin on DC-induced T-cell differentiation, MLR experiments were performed with a 1:10 ratio of DCs to T cells. The flow cytometry results showed that, compared with unstimulated T cells, DCs could strongly promote CD4^+^ CD25^+^ Foxp3^+^ T_reg_ differentiation, especially for Met-mDCs. (Fig. 2d). Moreover, the expression levels of some immunomodulatory molecules in DCs, including ICOSL and PD-L1/2, were dramatically upregulated by metformin (Supplemental Fig. 2b). These results indicated that metformin could disturb LPS-induced DC maturation and shift these cells into a tolerant phenotype.

### Metformin remodeled multiple metabolic pathways in DCs

After antigen stimulation (such as lipopolysaccharide (LPS)), the levels of glycolysis, the tricarboxylic acid cycle (TAC) and OXPHOS, as well as anabolism in DCs were significantly enhanced to meet the needs of their maturation and immune functions [35, 49]. In the present study, Kyoto Encyclopedia of Genes and Genomes (KEGG) pathway enrichment analysis of differential metabolites from untargeted metabolomics analysis revealed that multiple metabolic pathways were altered in Met-mDCs compared to mDCs (Fig. 2e), including arginine biosynthesis, FAS, nitrogen metabolism, D-glutamine and D-glutamate metabolism and so on. It has been shown that arginine is increased in tolDCs and its metabolism confers immunosuppressive properties on DCs [50, 51]. Consistently, we found arginine biosynthesis was enhanced in Met-mDCs, suggesting that metformin could induce DCs into a tolerant phenotype. Other three metabolic pathways reflect FA and amino acid metabolism playing important regulatory roles on the immune phenotypes and functions of DCs, which will be discussed in detail thereinafter. Furthermore, it is interested that there were few metabolic pathway changes between imDCs and Met-mDCs (Supplemental Fig. 3a), and these cells had similar metabolic transformations compared with mDCs (Fig. 2e, Supplemental Fig. 3b). These results indicated that similar intracellular biological responses occurred in imDCs and Met-mDCs, suggesting that the latter may partially maintain some immune functions of imDCs.

### Metformin disturbed the balance in FA metabolism and increased the accumulation of FAs in DCs

**Fig. 3:**
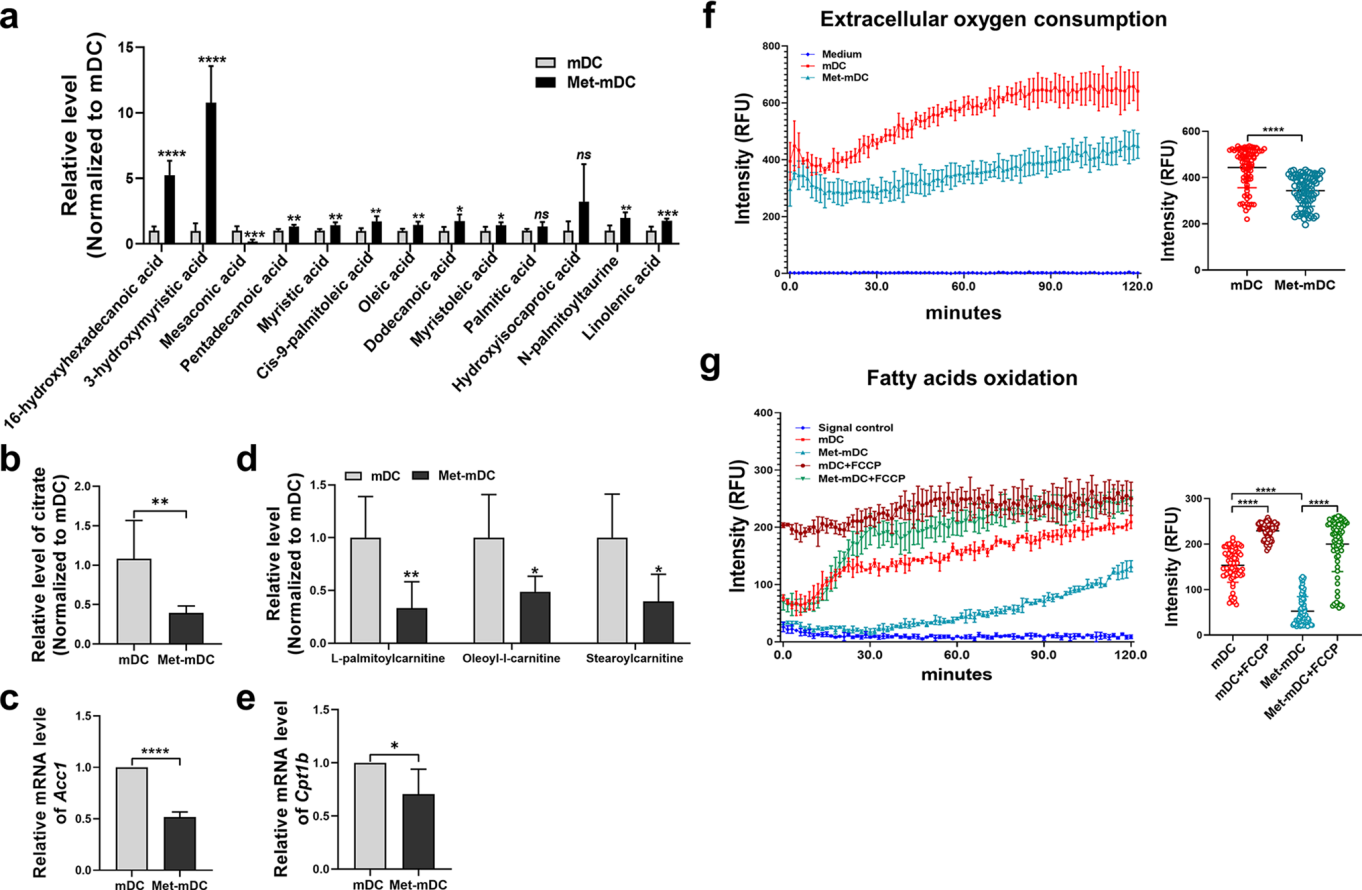
Metformin increased FA accumulation by disturbing the balance of FAS and FAO in DCs. **a** Related levels of FAs in mDCs and Met-mDCs were detected by untargeted metabolic analysis. N = 6. **b** Related levels of citrate in mDCs and Met-mDCs were detected by untargeted metabolic analysis. N = 6. **c** The expression level of *Acc1* was detected by RT‒qPCR. N = 6. **d** Related levels of carnitines in mDCs and Met-mDCs were detected by untargeted metabolic analysis. N = 6. **e** The expression level of *Cpt1b* was detected by RT‒qPCR. N = 6. **f** An extracellular oxygen consumption assay kit was used to measure the oxygen consumption of mDCs and Met-mDCs according to the manufacturer’s instructions, cell-free wells as the blank control. N = 3. **g** An FAO kit combined with an extracellular oxygen consumption assay kit were used to measure the level of FAO in mDCs, mDCs + FCCP (FAO agonist), Met-mDCs, Met-mDCs + FCCP (FAO agonist) according to the manufacturer’s protocols, cell-free wells as signal control. N = 3. Paired Student’s t tests were used for two-group comparisons; One-way ANOVA was used for multiple group comparisons. Mean ± SD *p < 0.05; **p < 0.01; ***p < 0.001; ****p < 0.0001

FAS is important for increasing endoplasmic reticulum and Golgi complex size in activated DCs, which is involved in protein synthesis and secretion [52]. However, excessive FA accumulation in the cell may play a pivotal role in maintaining the tolerogenic phenotype of DCs [53, 54]. In the present study, untargeted metabolomics analysis revealed that more FAs were present in Met-mDCs than in mDCs (Fig. 3a), indicating that metformin may increase FAS in DCs. Interestingly, citrate and acetyl-CoA carboxylase 1 (*Acc1*), which are the major factor and key enzyme for FAS, respectively, were significantly reduced (Fig. 3b, c), suggesting that the degree to which FAs were elevated in Met-mDCs might be independent of FAS. Further analyses showed that the levels of carnitines such as l-palmitoylcarnitine, oleoyl-l-carnitine and stearoylcarnitine were decreased in Met-mDCs (Fig. 3d). Carnitines are vital transporters of medium-long chain FAs into mitochondria to participate in FAO, and they perform crucial roles in the catabolism of FAs [55, 56]. Carnitine palmitoyltransferase 1 (*Cpt1*), an important FAO enzyme, was also downregulated in Met-mDCs (Fig. 3e). This evidence suggested that metformin inhibited FAO in DCs. Furthermore, the results of the present study revealed that oxygen consumption and the level of FAO in Met-mDCs were significantly decreased (Fig. 3f, g). Therefore, metformin may disrupt the balance in FAS and FAO, ultimately leading to excess FA accumulation in Met-DCs. In addition, we also noticed that there were higher levels of FAs in imDCs compared to mDCs, while the increasing trend was moderate (Supplemental Fig. 4a). It has been indicated that imDCs with dominated catabolism could employed OXPHOS (such as FAO) to gain enough energy to meet the need of their own life activities and functions [35]. Consistently, our results revealed that imDCs were with highest levels of carnitines, compared with mDCs and Met-mDCs (Supplemental Fig. 4b), suggesting enhanced FAO occurred in imDCs by which they expend the FAs derived from catabolism of other substance and ensure the proper levels of FAs and functions of imDCs. As a result, although increasing levels of FAs were both detected in imDCs and Met-mDCs, there was pathological accumulation of FAs within Met-mDCs due to the inhibition of FAO, which led to alterations of DC’s immuneophenotypes and functions.

### Metformin promoted excess lactic acid production by rewiring glycolysis and the TAC in DCs

**Fig. 4:**
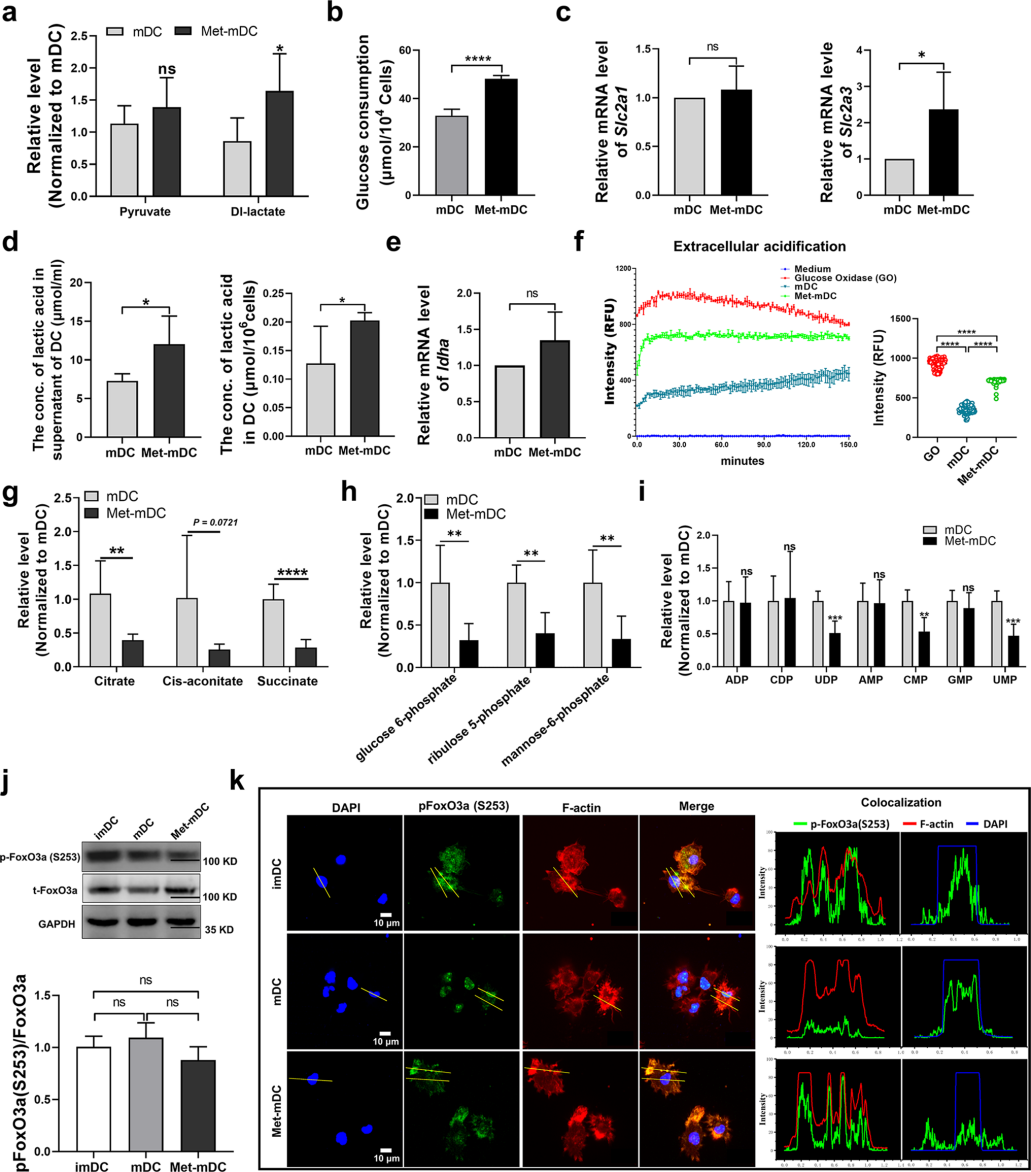
Metformin promoted lactic acid production by disturbing glycolysis, the TAC and the PPP in DCs and inhibited the nuclear translocation of pFoxO3a. **a** Related levels of pyruvate and lactate in mDCs and Met-mDCs were detected by untargeted metabolic analysis. N = 6. **b** Glucose concentrations in the supernatant of cultured mDCs and Met-mDCs were detected by a glucose measuring kit, and then glucose consumption was calculated by the following formula: (initial glucose concentration—measured glucose concentration) × volume (600 μl). N = 6. **c** The expression levels of GLUTs (*slc2a1 and slc2a3*) were detected by RT‒qPCR. N = 4. **d** The levels of lactate in DCs and in their culture supernatant were detected using a lactate assay kit. N = 6. **e** The expression level of *ldha* was detected by RT‒qPCR. N = 5. **f** The extracellular acidification, which reflects cellular glycolytic flux, was measured using a glycolysis assay kit. mDCs and Met-mDCs were prepared according to the manufacturer’s instructions, and glycolysis signals were detected with Cytation 5. N = 3. **g**, **h** and **i** Related levels of the intermediates of the TAC (**g**), glycolysis and the PPP (**h**), as well as nucleotides (**i**) in mDCs and Met-mDCs, were detected by untargeted metabolic analysis. N = 6. **j** The levels of pFoxO3a (S253) and FoxO3a in imDCs, mDCs and Met-mDCs were detected by western blotting. N = 4. **k** The localization of pFoxO3a (S253) in imDCs, mDCs and Met-mDCs was analyzed by a laser scanning confocal microscope. From left to right are representative confocal images of nuclei (blue), pFoxO3a (S253) (green), F-actin (red), merged fluorescence images and colocalization analysis of pFoxO3a. Paired Student’s t tests were used for two-group comparisons. One-way ANOVA was used for comparisons among three groups. Mean ± SD *p < 0.05; **p < 0.01; ***p < 0.001; ****p < 0.0001

Glucose metabolism is one of the three major metabolic pathways that supply energy and intermediates for cell survival and function. By examining the differential metabolites between Met-mDCs and mDCs, we found that there were relatively higher levels of pyruvate and lactate in Met-mDCs (Fig. 4a), suggesting that metformin could affect glucose metabolism in DCs. As expected, the glucose consumption assay results showed that Met-mDCs had a higher glucose uptake capacity (Fig. 4b) than mDCs. Furthermore, Met-mDCs had elevated expression levels of the glucose transporter (*Slc2a3*) (Fig. 4c), increased lactate production (Fig. 4d), higher expression of lactate dehydrogenase A (*Ladha*) (Fig. 4e) and higher cellular glycolytic flux (Fig. 3f) than mDCs. These data suggested that metformin could promote glycolysis in DCs. However, it seems that TAC, the next metabolic pathway following glycolysis, was not strengthened by metformin because the data showed that some TAC intermediates, including citrate, cis-aconitate and succinate, were dramatically reduced in Met-mDCs (Fig. 4g), suggesting that metformin could inhibit TAC in DCs and inhibit pyruvate entry into the TAC, resulting in lactate accumulation in DCs, which has been shown to be a negative regulatory factor for DC functions [57, 58]. Furthermore, the levels of these TAC intermediates in Met-mDCs were lower than in imDCs (Supplemental Fig. 4c), indicating that metformin seriously inhibited the activities of TAC in Met-mDCs rather than simply retaining low level of TAC similar as in imDCs. Taken together, these results demonstrated that metformin could inhibit the metabolic reprogramming of DCs mediated by LPS and the resulting metabolic disorder (promoted glucose consumption and glycolysis but inhibited TAC) led to excess lactic acid accumulation in DCs and skewed them toward to a tolerant phenotype.

### Metformin inhibited the PPP and maintained partial catabolic activity in DCs

The untargeted metabolic analysis showed obvious declines in ribulose-5-phosphate (R5P) and mannose-6-phosphate (M6P), which are intermediates of the PPP, in Met-mDCs (Fig. 4h), indicating that metformin inhibited the PPP during LPS-induced DC maturation. R5P is a raw material for nucleotides. As shown in Fig. 4i, the levels of several nucleotides, including cytidine 5'-monophosphate (CMP), uridine 5'-monophosphate (UMP) and uridine 5'-diphosphate (UDP), were decreased in Met-mDCs, which might be caused by insufficient R5P. In addition, anabolism is a major mechanism by which mDCs synthesize proteins to meet their functional needs. Amino acids are the main materials for protein synthesis that are also decomposed from proteins. As shown in supplemental Fig. 4d, Met-mDCs and imDCs had more free amino acids and related decomposed intermediates than mDCs, suggesting that, exactly as imDCs, Met-mDCs may maintain partial catabolic activity. These results demonstrated that metformin could inhibit the PPP and partially conserve catabolism in DCs, which likely resulted in reduced biosynthesis in DCs.

### Metformin rearranged the localization of pFoxO3a in DCs

KEGG pathway enrichment analysis indicated that the FoxO signaling pathway was activated in imDCs and Met-mDCs (Fig. 2e, Supplemental Fig. 3b). It is well known that FoxO transcription factors play important roles in multiple cellular processes, such as metabolism, the immune response, cell survival, aging and longevity [59]. FoxO3 is one FoxO subset and is particularly involved in cell metabolism and immune plasticity [60]. Upregulation of FoxO3 could lead to tolerogenic properties in DCs [61]. Thus, it could be hypothesized that FoxO3 is controlled by metformin in the regulation of DC functions. Ser253 is an important residue that promotes FoxO3 translocation into the nucleus after it is phosphorylated, which mediates the expression of many FoxO3-targeted genes [62]. In the present study, there was lower expression of pFoxO3 (S253) in imDCs and Met-mDCs than in mDCs, but unfortunately no significant statistical difference among these three (Fig. 4j), and also no difference of pFoxO3 (S253) between mDCs and Met-mDCs from the results of immunofluorescence images (Supplemental Fig. 5). However, further analysis revealed that pFoxO3a of mDCs was mainly localized in nucleus while in cytoplasm for Met-mDCs, as well as more pFoxO3a of imDCs was biased to localized in cytoplasm (Fig. 4k), suggesting that pFoxO3a was relocated in response to LPS and/or metformin stimulation. LPS promoted pFoxO3a entry into the nucleus, while metformin shuttled it back to the cytoplasm, indicating that metformin could regulate the activation of DCs through the FoxO3a signaling pathway.

Based on these in vitro results, we inferred that during LPS-induced DC maturation, metformin could dramatically affect multiple metabolic pathways in DCs. The balance between FAS and FAO were disturbed, DC glucose consumption and glycolysis were increased, the TAC and PPP were inhibited, and partial catabolism in DCs was maintained, leading to the accumulation of FAs and lactic acid in DCs and remodeling DC immunophenotype and functions. FoxO3a are likely the targets by which metformin regulates these metabolic events in DCs.

### Metformin played strong anti-inflammatory roles in vivo by shaping the immunophenotype of DCs

**Fig. 5:**
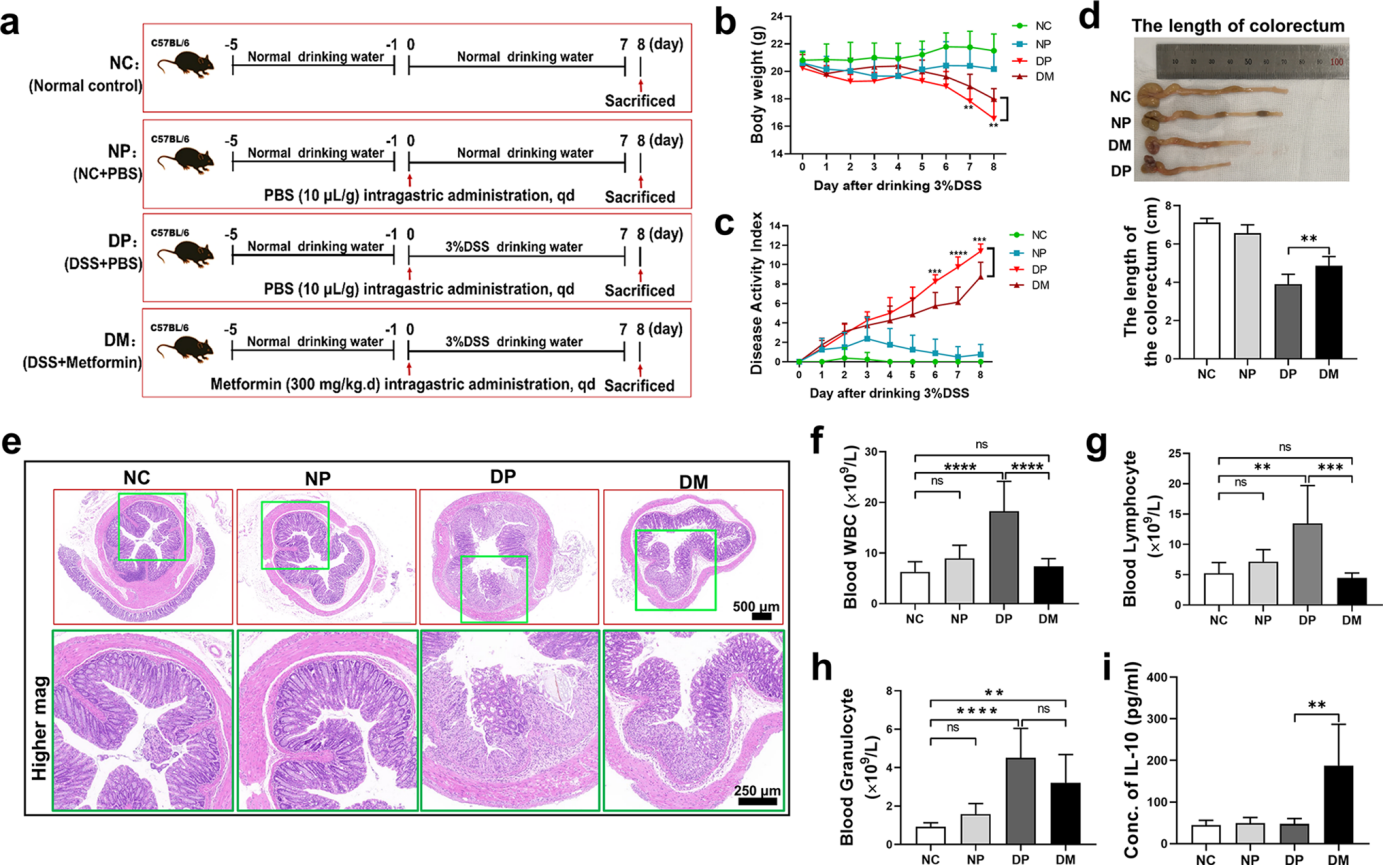
Metformin played anti-inflammatory roles in vivo and attenuated the clinical symptoms of mice with IBD. **a** Diagram of model construction and the treatment of IBD. **b** Body weight changes in mice in the NC, NP, DP and DM groups. N = 8. **c** To determine the disease activity index, the total score was calculated based on the weight loss, hematochezia and stool consistency of mice from the NC, NP, DP and DM groups; a higher score corresponded to colitis severity. N = 8. **d** The length of the colorectum of mice in the NC, NP, DP and DM groups. N = 6. **e** Representative H&E-stained colorectal sections of mice in the NC, NP, DP and DM groups. **f**, **g**, **h** The white blood cell (**f**), lymphocyte (**g**), and granulocyte (**h**) counts in the peripheral blood of mice in the NC, NP, DP and DM groups. N = 6. **i** The concentration of IL-10 in the peripheral blood of mice in the NC, NP, DP and DM groups. N = 4. Unpaired Student’s t tests were used for two-group comparisons. One-way or two-way ANOVA was used for comparisons of three groups. Mean ± SD *p < 0.05; **p < 0.01; ***p < 0.001; ****p < 0.0001

**Fig. 6:**
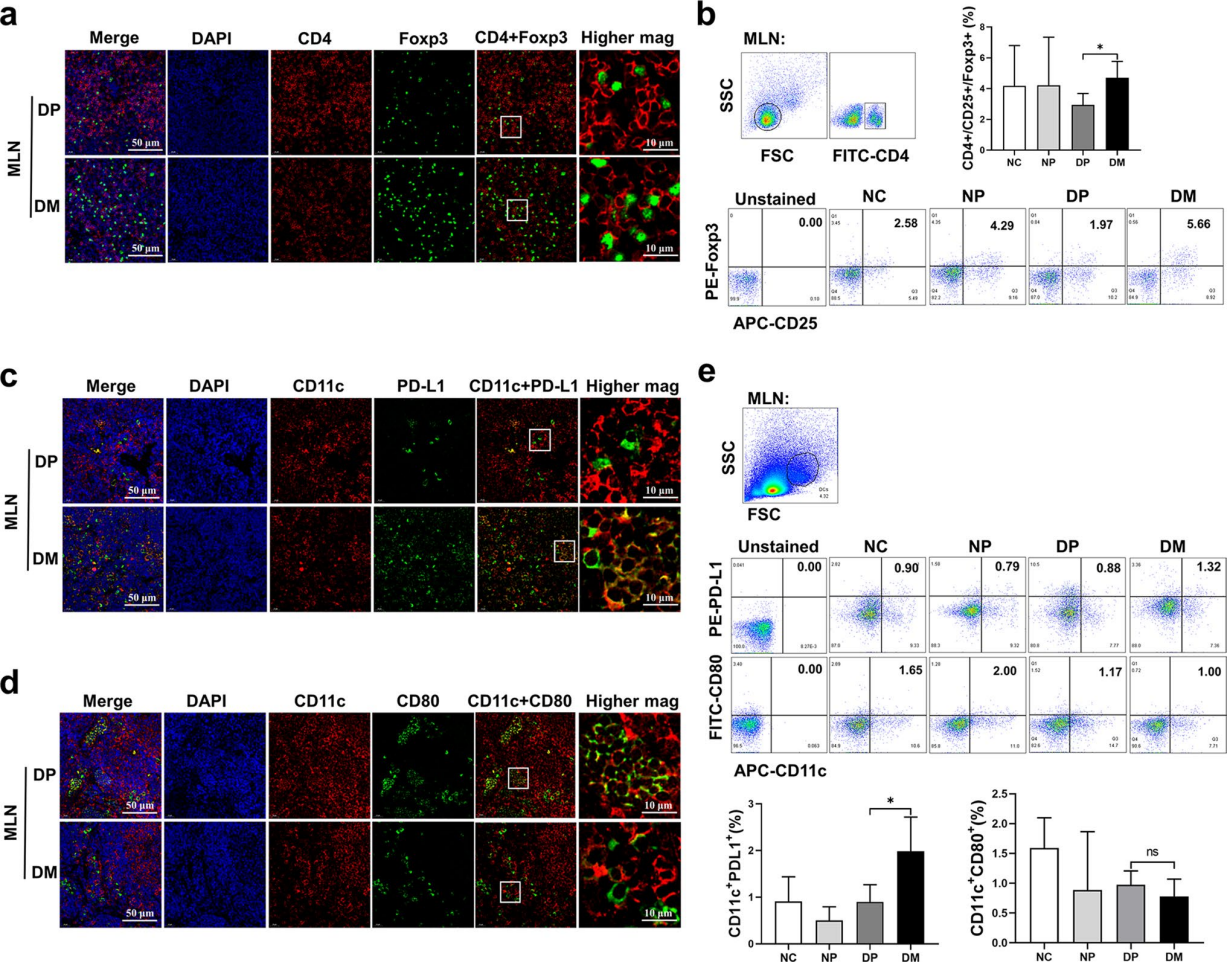
Metformin promoted the proportion of T_regs_ in MLNs, upregulated the expression of PD-L1 and reduced the expression of CD80 in MLN-derived DCs from the mice with IBD. **a**, **c**, **d** Representative fluorescent images of CD4, Foxp3, CD11c, PD-L1 and CD80 in MLN cells from mice in the DP and DM groups. From left to right are images of the four channels merged, nuclei (blue), CD4/CD11c (red), Foxp3/PD-L1/CD80 (green), double channels merged and partial enlarged images, respectively. **b** The proportion of T_regs_ among the MLN cells of mice in the NC, NP, DP and DM groups was measured by flow cytometry. N = 5. **e** The expression levels of PD-L1 and CD80 in MLN DCs from mice in the NC, NP, DP and DM groups were measured by flow cytometry. N = 6. Unpaired Student’s *t* tests were used for two-group comparisons. Mean ± SD *p < 0.05; **p < 0.01

To further elucidate the anti-inflammatory roles of metformin, AD mouse models of IBD were developed and induced into ulcerative colitis (UC) with 3% DSS (Fig. 5a). Compared with those in the DP group (sick animals that received PBS treatment as a control), mice in the DM group (sick animals that received metformin treatment) had less weight loss (Fig. 5b) and disease activity indices (Fig. 5c), longer colorectal lengths (Fig. 5d), and less intestinal mucosal damage (Fig. 5e), suggesting that metformin could significantly improve the clinical symptoms of IBD. Furthermore, metformin reduced leukocyte, lymphocyte and granulocyte counts in the peripheral blood of sick mice (Fig. 5f, g, h), increased the serum levels of IL-10 (Fig. 5i) and increased the infiltration of T_regs_ in the MLN (Fig. 6a, b) and spleen (Supplemental Fig. 6a, b). These results indicated that metformin played strong anti-inflammatory roles in vivo. Evidence has shown that DCs exhibit an obvious mature status with high expression of costimulatory molecules and MHC-II, excessive secretion of proinflammatory cytokines, and a strong ability to initiate effector T cells in hosts with IBD, promoting the development of the disease [63–65]. To confirm that the anti-inflammatory roles of metformin were associated with its effects on the DC immunophenotype, CD11c^+^ DCs from DP and DM group mice were analyzed by immunofluorescence microscopy (IFM) and flow cytometry. The data revealed that the expression levels of PD-L1 on DCs in the MLNs (Fig. 6c, e above) and spleens (Supplemental Fig. 6c, d) of metformin-treated mice were significantly increased. PD-Ls are critical immune checkpoint molecules that play crucial roles in mediating T_reg_ differentiation or inducing T-cell exhaustion by conjugating with their ligand PD-1 to enhance immune tolerance [66, 67]. Thus, the overexpression of PD-L1 caused by metformin in DCs must be one of the reasons that metformin increased the number of T_regs_ in vivo. In addition, although there was no difference in CD80 as measured by flow cytometry between the DP and DM groups (Fig. 6e below), the IFM results showed that metformin could decrease the expression of CD80 on DCs (Fig. 6d), suggesting that metformin may also impair the antigen presentation ability of DCs in vivo. In addition, to further validate the anti-inflammatory effect of metformin is directly related to DCs, we performed DCs-based cell therapy for mice with IBD (Supplemental Fig. 7a). Compared with the groups which the mice received treatment with PBS or mDCs intraperitoneal injection, mice with IBD that received treatment with imDCs and Met-mDCs had less weight loss (Supplemental Fig. 7b), less intestinal mucosal damage (Supplemental Fig. 7c) and longer colorectal lengths (Supplemental Fig. 7d, e). Moreover, the counts of leukocyte, lymphocyte and granulocyte from peripheral blood of sick mice treated with imDCs and Met-mDCs were obviously decreased (Supplemental Fig. 7f–h). These findings suggested that metformin-modified DCs possessed anti-inflammatory roles and could significantly improve the clinical symptoms of IBD. Therefore, metformin may modulate the immunophenotype and functions of DCs to exert its anti-inflammatory effects in vivo.

**Fig. 7:**
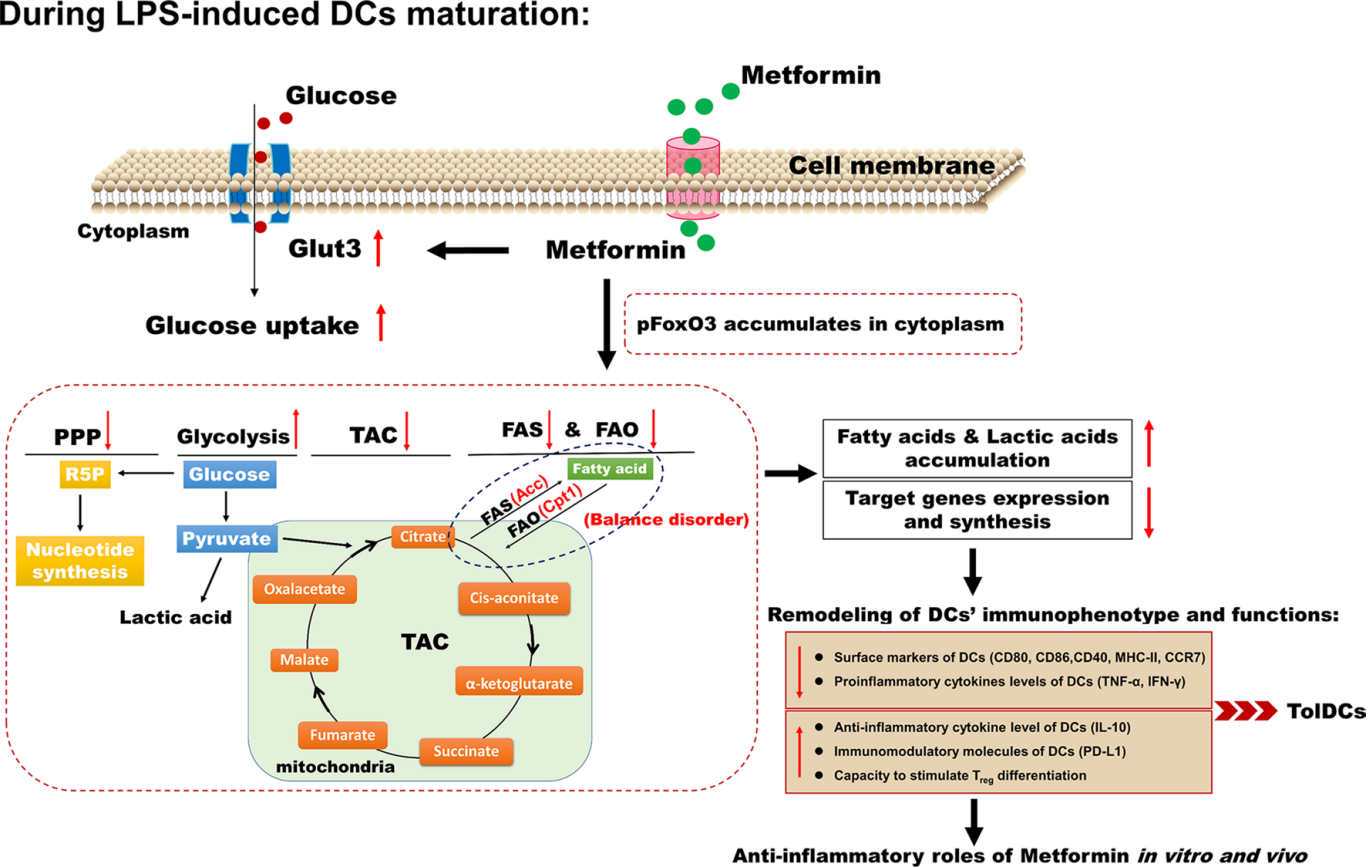
Schematic illustration of the effects of metformin on DC metabolic reprogramming and function. *Glut3* glucose transporter3, *PPP* pentose phosphate pathway, *TAC* tricarboxylic acid cycle, *FAS* fatty acid synthesis, *FAO* fatty acid oxidation, *Acc* acetyl-CoA carboxylase, *Cpt1* Carnitine palmitoyltransferase 1, *pFoxo3a* phosphorylated forkhead box-O3a

Taken together, metformin could induce tolerogenicity of DC by promoting metabolic reprogramming and play anti-inflammatory role both in vitro and in vivo. A schematic representation of the effects of metformin on DCs is shown in Fig. 7.

## Discussion

Because of the special roles of DCs in bridging innate and adaptive immunity and their bilateral functions of immune response and tolerance, DC has become the logical target for the treatment of immune-mediated diseases. Remodeling of DC’s immunophenotype and functions can alter the immune response type of microenvironment in organs or tissues, which inhibit the immunopathological progression of disease (like ADs) or enhance the anti-tumor immunity. Recently, emerging evidence indicated that metformin played notable immunomodulatory roles, suggesting a novel perspective that metformin may be a preferable candidate drug for patients with immune-mediated disease, especially ADs, because of its anti-inflammatory properties [68]. To date, it has been found that restoring the balance between effector T cells (Th_17_) and T_regs_ [22, 23], inhibiting the transformation of B cells into plasma cells and related autoantibody production [69, 70], guiding macrophage polarization to the M_2_ subset [71] and reducing the secretion levels of proinflammatory cytokines [24, 25] are the main mechanisms by which metformin restrains the pathological progression of ADs. However, whether the therapeutic effectiveness of metformin in ADs is related to its regulatory roles on the immunophenotype and functions of DCs were unclear. Seulmee et al. reported that metformin could suppress DC MHC-restricted antigen presentation capabilities by inhibiting costimulatory factors and MHC molecules [72], but the underlying mechanisms are still elusive. In the current study, we revealed that metformin could abrogate the elevated expression levels of MHC-II and costimulatory molecules induced by LPS (Fig. 1a, b) and weaken DC T-cell activation (Fig. 2a). Metformin also significantly attenuated DC chemotaxis (Fig. 2b) and rescued their endocytic abilities (Fig. 2c). These results indicated that metformin inhibited the maturation of DCs under inflammatory conditions. Fully mature DCs can effectively induce adaptive immune responses; in contrast, imDCs and semi-mDCs are more likely to induce T-cell anergy and immune tolerance [1]. Thus, the anti-inflammatory effects of metformin also involved inhibiting DC maturation and functions.

Furthermore, metformin altered the secreted cytokine profiles of DCs (Fig. 1c), downregulating proinflammatory cytokines (TNF-α and IFN-γ) and increasing the levels of IL-10, which are required for effector T-cell and T_reg_ differentiation, respectively, and decreasing the secretion of TGF-β, which is vital for Th_17_ polarization [4]. Cytokines provide the third signal for T-cell activation, which directs naïve T-cell polarization into appropriate subsets, modifying different types of immune responses [4]. As expected, Met-mDCs exhibited a powerful capacity to induce T_reg_ differentiation and proliferation in the current study (Fig. 2d). Furthermore, metformin dramatically upregulated the expression levels of immunomodulatory molecules (ICOSL, PD-Ls) (Supplemental Fig. 2b), which are representative markers of tolDCs and are essential for DC-mediated induction of tolerogenic responses [9]. Further the present study showed that metformin played anti-inflammatory roles in vivo (Fig. 5) by shifting DCs to a phenotype with a high expression level of PD-L1 (Fig. 6c, e above) and reducing the expression level of CD80 (Fig. 6d, e below). Moreover, metformin-modified DCs played anti-inflammatory roles and improved the clinical symptoms of mice with IBD (Supplemental Fig. 7). These results suggest that metformin skews DCs toward a tolerant phenotype. TolDCs are responsible for establishing and maintaining the immune homeostasis of the body [73]. Taking advantage of the features of tolDCs can suggest promising therapeutic regimens for treating ADs. Thus, these results further provide favorable evidence for the use of metformin in ADs.

It is clear that metabolic reprogramming is closely linked to immune cell polarization and affects immune functions [28]. Studies have shown that different metabolic patterns occur in DCs at different stages of differentiation [35]: catabolism is dominantly used by imDCs to generate energy from glycogen, glutamine, FAs, etc., through the β oxidation and OXPHOS, while mDCs have enhanced intracellular glycolysis and the PPP and TAC to acquire energy and metabolic intermediates for biosynthesis, indicating that metabolic reprogramming contributes to the functional plasticity of DCs. The mechanisms of the pharmacological effects of metformin contribute to its vigorous regulatory roles in cellular metabolism, including that of glycose, FAs and amino acids [40]. Here, we explored the mechanisms by which metformin shapes DC immunophenotype and functions from the perspective of immune metabolism. As expected, multiple metabolic pathways were altered by metformin during LPS-induced DC maturation and the KEGG pathway enrichment analysis revealed that many similar metabolic patterns presented in imDCs and Met-mDCs (Fig. 2e and Supplemental Fig. 3a, b). But as stated in results section, there were difference of some metabolic pathways in detail between imDCs and Met-mDCs, such as FAO and TAC, which indicated that instead of simply stagnating DCs in immature state, metformin had complex regulatory roles on DCs’ metabolic patterns and further remodeled their phenotypes and functions.

Unlike slightly elevated FAs within imDCs, redundant FAs were abnormally accumulated in Met-mDCs (Fig. 3a and Supplemental Fig. 4a). It is well known that appropriate levels of FAS and FAs are pivotal for DC maturation and activation [74]. Patricia et al. demonstrated that inhibiting FAS by a-fetoprotein (AFP) intensively impaired the capacity of DCs to stimulate antigen-specific effector functions [75]. In contrast, lipids accumulated in DCs when they were exposed to a high FA levels in the tumor microenvironment, markedly disabling the immune priming capacity of DCs [54]. Another recent study confirmed that vitamin D (1,25-dihydroxyvitamin D; 1,25D) induced tolDC generation by promoting the FAS pathway [53]. These observations indicate that FAS is indispensable for DCs, while excess FA accumulation appears to suppress DC immune functions and modulates these cells to a tolerant phenotype. The present study revealed that metformin led to the marked accumulation of FAs in DCs because of the disrupted balance between FAS and FAO (Fig. 3). Based on these results, it appears reasonable that the resulting FA accumulation is a mechanism by which metformin skews DCs toward a tolerant phenotype.

The results also showed that metformin could increase the expression level of GLUT (*slc2a3*) in DCs (Fig. 4c) and elevate the use of glucose during LPS-induced DC maturation (Fig. 4b). Subsequent data from the glycolysis assay revealed that there was higher cellular glycolytic flux in metformin-treated mDCs (Fig. 4f), suggesting that metformin could enhance the intracellular glycolysis of DCs, which was also verified by the fact that higher levels of pyruvate and excess production of lactate were detected in Met-mDCs in the untargeted metabolic analysis (Fig. 4a, d). However, as shown in the present study, Met-mDCs with a tolerant phenotype should not have excessive glycolysis, according to the evidence that there was lower glucose consumption and glycolytic response in imDCs and tolDCs [35]. Further analysis of the untargeted metabolic analysis data showed that the levels of several intermediates (citrate, cis-aconitate and succinate) in the TAC were reduced in Met-mDCs (Fig. 4g), suggesting that the TAC was defective in these cells. It is therefore possible that although metformin promoted glycolysis, it inhibited the TAC during LPS-induced DC maturation, leading to lactic acid production and accumulation in the cells. Recent studies indicate that lactic acid can mediate immune cell functions [57]. Lactate can be transported into DC cytoplasm and profoundly stimulate DCs to an anti-inflammatory state [76]**,** resulting in lower expression levels of MHC-II and costimulatory molecules, decreased secretion of IL-12, higher levels of IL-10, defective migration and the upregulation of immunomodulatory molecules (RA, IDO, etc.). Our previous study demonstrated that lactate could damage the motility and mechanical properties of DCs by disturbing cytoskeletal remodeling [77]. A recent study showed that the production of excess lactic acid was an important mechanism by which tolDCs shape T-cell responses toward tolerance [78]. As a result, it is likely that promoting lactic acid accumulation in DCs by disrupting glucose metabolism (increasing glycolysis and decreasing TAC) is a major mechanism by which metformin remodels DC immunophenotype and functions.

In addition, we noticed that the levels of M6P and R5P, the metabolic intermediates of the PPP, were decreased in Met-mDCs (Fig. 4h), suggesting that the PPP was inhibited by metformin. R5P is the principal material for nucleotide synthesis and RNA and protein synthesis [79]. Because of a lack of R5P, the levels of several nucleotides were decreased, which could cause the low biosynthesis in Met-mDCs (Fig. 4i). Moreover, metformin increased free amino acids and related decomposition intermediates in mDCs (Supplemental Fig. 4d), suggesting that it maintained partial catabolic activity in LPS-molecules. Overall, metformin could reduce anabolism while promoting catabolism in DCs, leading to the downregulation of immunophenotypic markers and decreased cytokine secretion.

Mechanistically, the findings revealed that the FoxO signaling pathway was activated in imDCs and Met-mDCs (Fig. 2e, Supplemental Fig. 3b). FoxOs belong to the Forkhead box protein family and plays important roles in cell proliferation, apoptosis, autophagy, oxidative stress and metabolic regulation [59]. Studies have demonstrated that the upregulation of FoxO3, a subset of FoxOs, could lead to tolerogenic properties in DCs and impair their ability to activate effector T cells [61]. FoxO3a, which is an important transcription factor, can promote the gene expression of MHC-II and costimulatory molecules in DCs when it enters the nucleus [80, 81]. Metformin reduced the expression level of pFoxO3a (Ser253) and relocated pFoxO3a in the cytoplasm during LPS-induced DC maturation (Fig. 4j, k). Thus, the inhibitory effect of metformin on the expression levels of DC immunophenotypic molecules and cytokines may be related to its inhibitory effects on the nuclear localization of pFoxO3. Taken together, these results suggest that FoxO3a signaling pathways are used by metformin to regulate metabolic reprogramming and the synthesis of important molecules in DCs and further remodel their immunophenotype and functions.

In conclusion, metformin could inhibit LPS-induced DC maturation and shift these cells into tolerant phenotypes, playing dramatic anti-inflammatory roles in vitro and in vivo. The underlying mechanisms were related to metformin-induced accumulation of FAs and lactic acid in DCs through disturbances in the balance of FAS and FAO, glycolysis, the TAC and the PPP, which are regulated by FoxO3a signaling pathways. Our findings reinforce the understanding of the immunoregulatory mechanisms of metformin and lay a foundation for the application of metformin in ADs, as well as other immune-mediated diseases.

### Supplementary Information

Below is the link to the electronic supplementary material.

Supplementary file1 (TIF 249 KB)

Supplementary file2 (TIF 310 KB)

Supplementary file3 (TIF 967 KB)

Supplementary file4 (TIF 486 KB)

Supplementary file5 (TIF 75 KB)

Supplementary file6 (TIF 3235 KB)

Supplementary file7 (TIF 1687 KB)

Supplementary file8 (DOCX 19 KB)

Supplementary file9 (DOCX 15 KB)

## Data Availability

All data supporting the findings from this study are available from the corresponding author or the first author upon reasonable request.
