# Supplementary figures and images for: Metformin induces tolerogenicity of dendritic cells by promoting metabolic reprogramming
Source: Cell Mol Life Sci. 2023 Sep 9;80(10):283. doi: 10.1007/s00018-023-04932-3 (PMC10492886; doi:10.1007/s00018-023-04932-3)

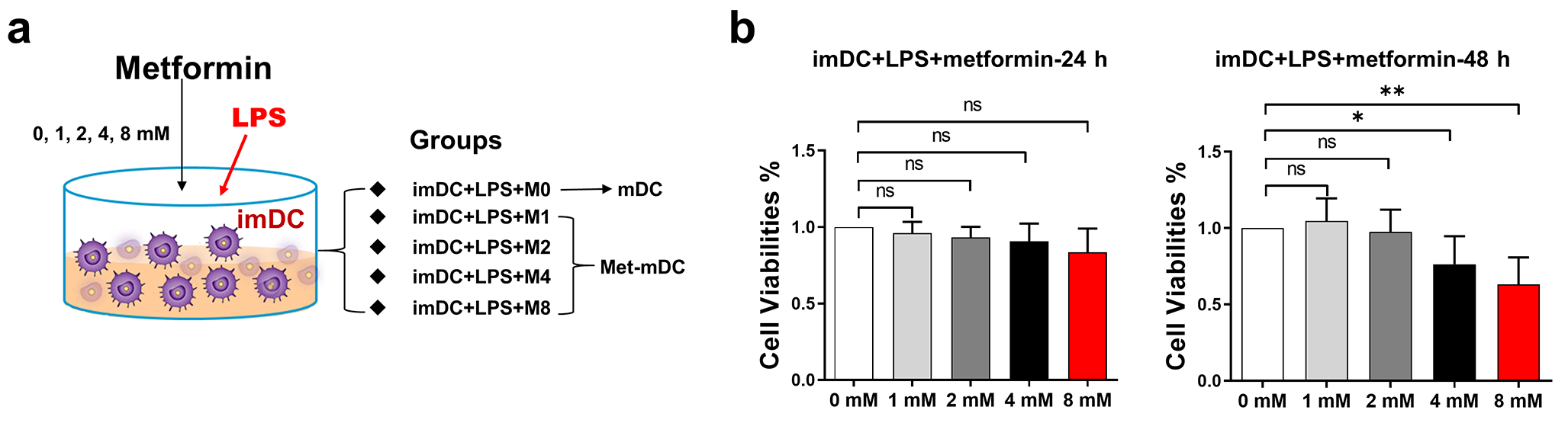

Supplement: Supplementary file 1 — Supplementary file1 (TIF 249 KB) [file 18_2023_4932_MOESM1_ESM.tif]

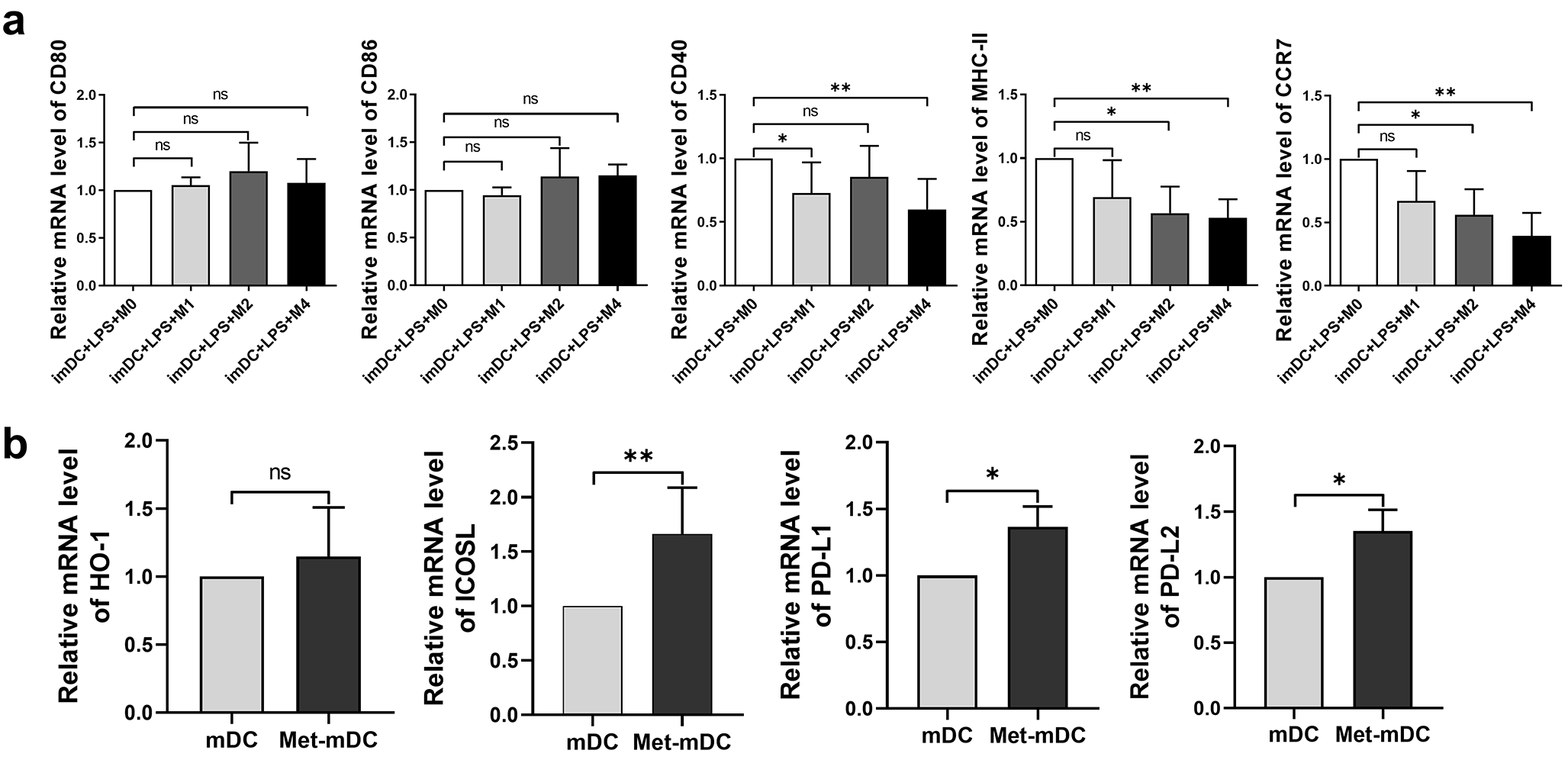

Supplement: Supplementary file 2 — Supplementary file2 (TIF 310 KB) [file 18_2023_4932_MOESM2_ESM.tif]

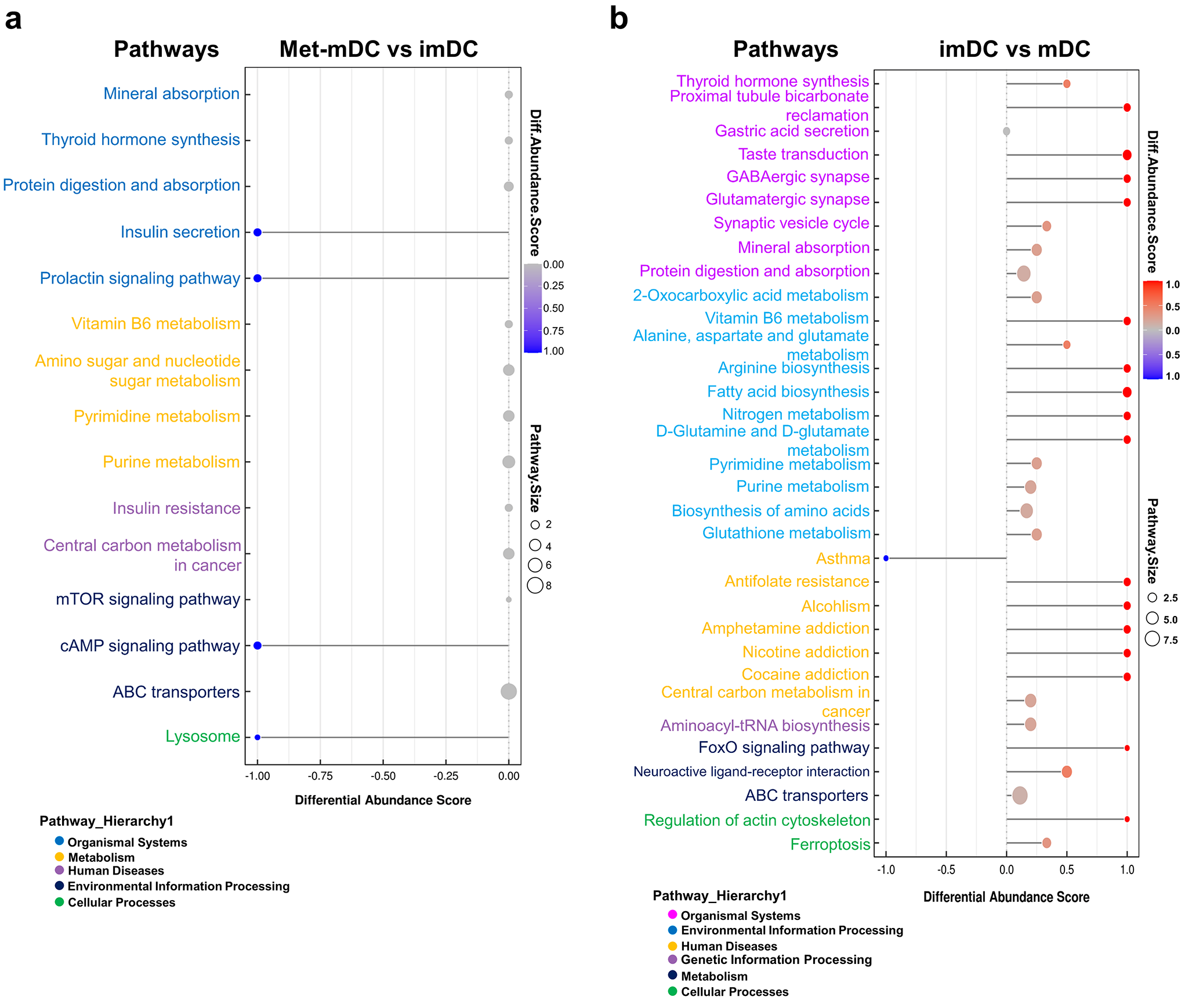

Supplement: Supplementary file 3 — Supplementary file3 (TIF 967 KB) [file 18_2023_4932_MOESM3_ESM.tif]

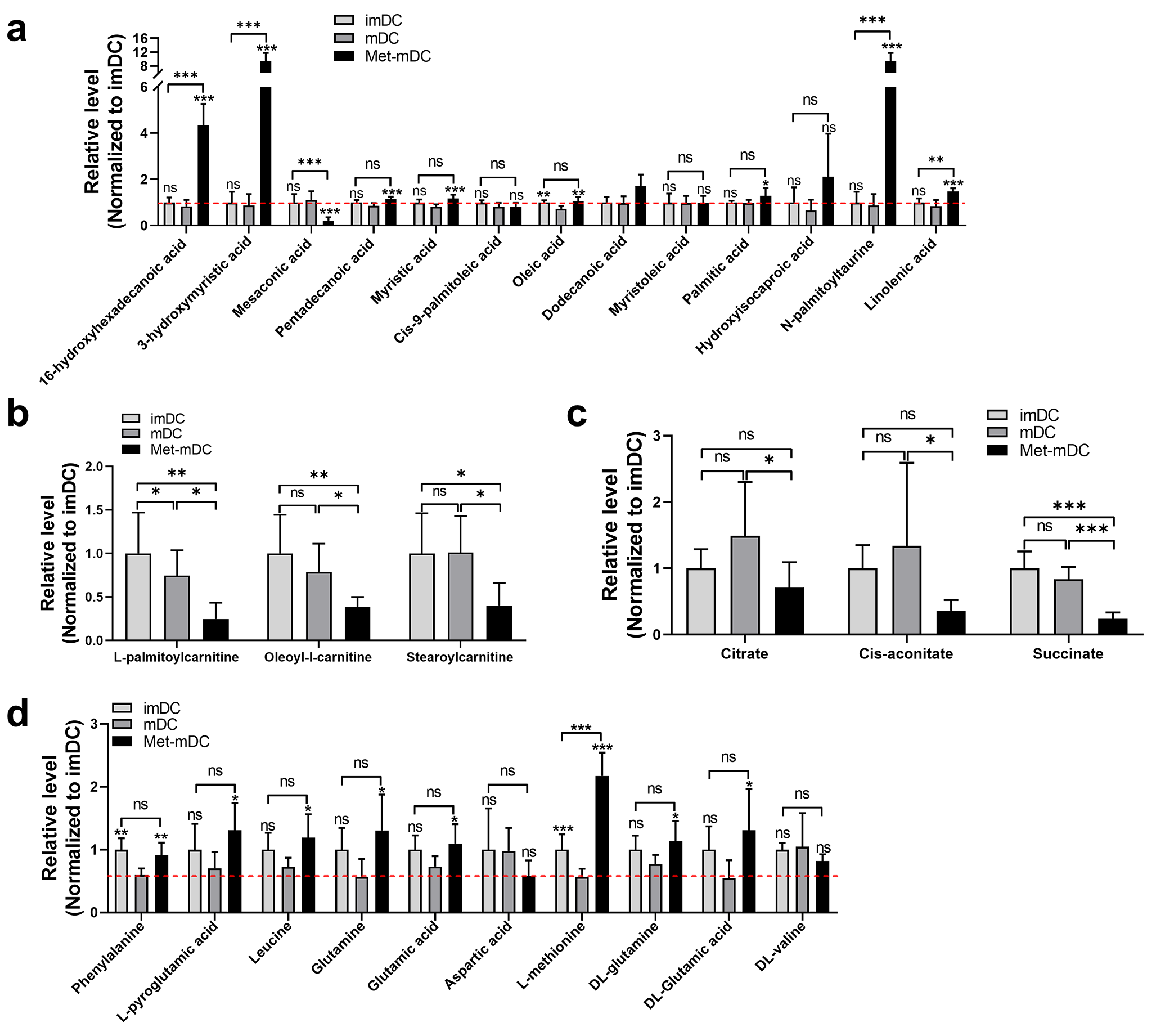

Supplement: Supplementary file 4 — Supplementary file4 (TIF 486 KB) [file 18_2023_4932_MOESM4_ESM.tif]

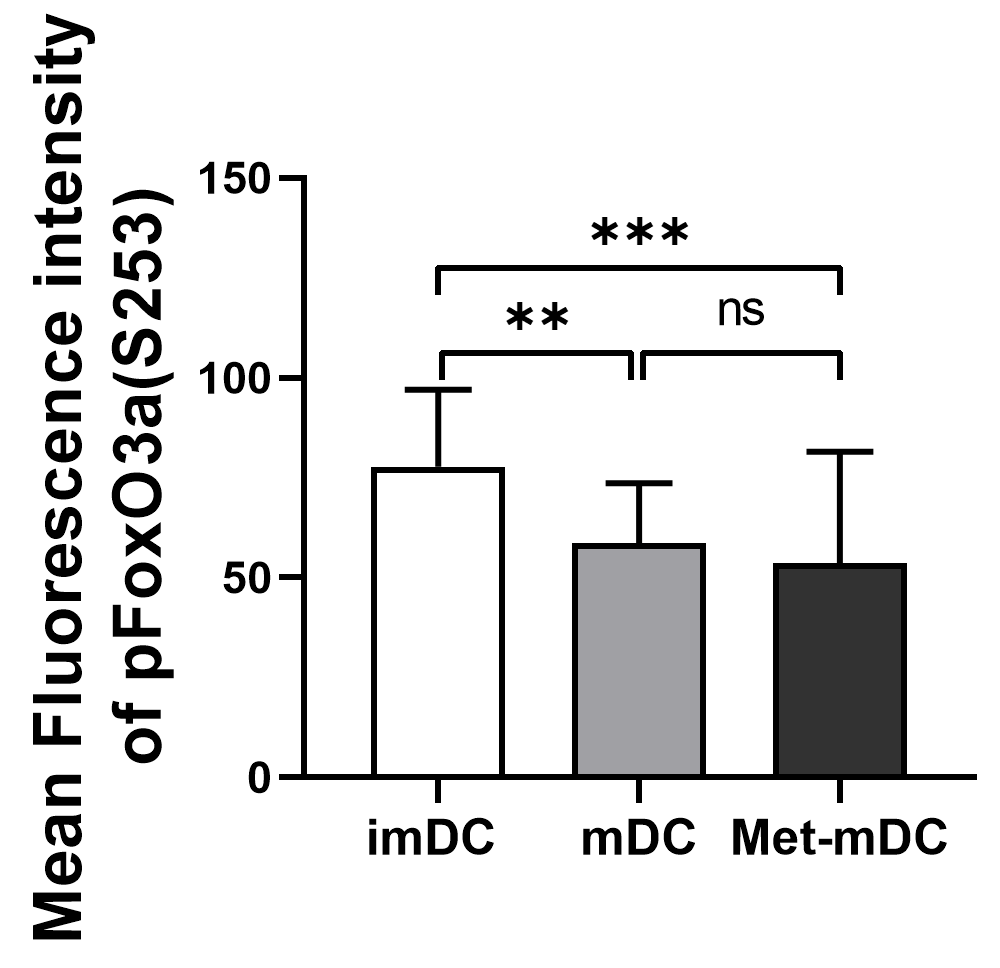

Supplement: Supplementary file 5 — Supplementary file5 (TIF 75 KB) [file 18_2023_4932_MOESM5_ESM.tif]

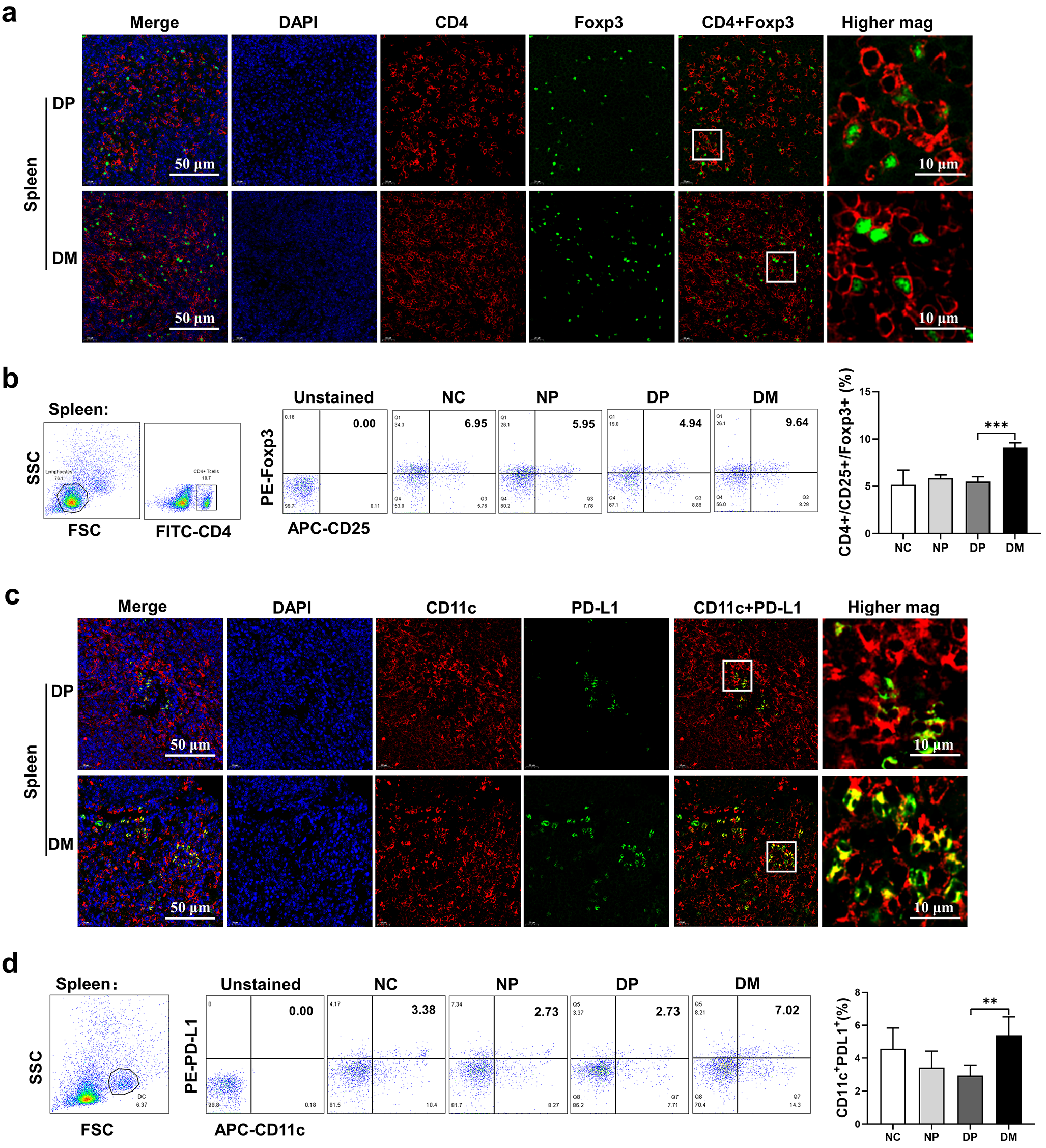

Supplement: Supplementary file 6 — Supplementary file6 (TIF 3235 KB) [file 18_2023_4932_MOESM6_ESM.tif]

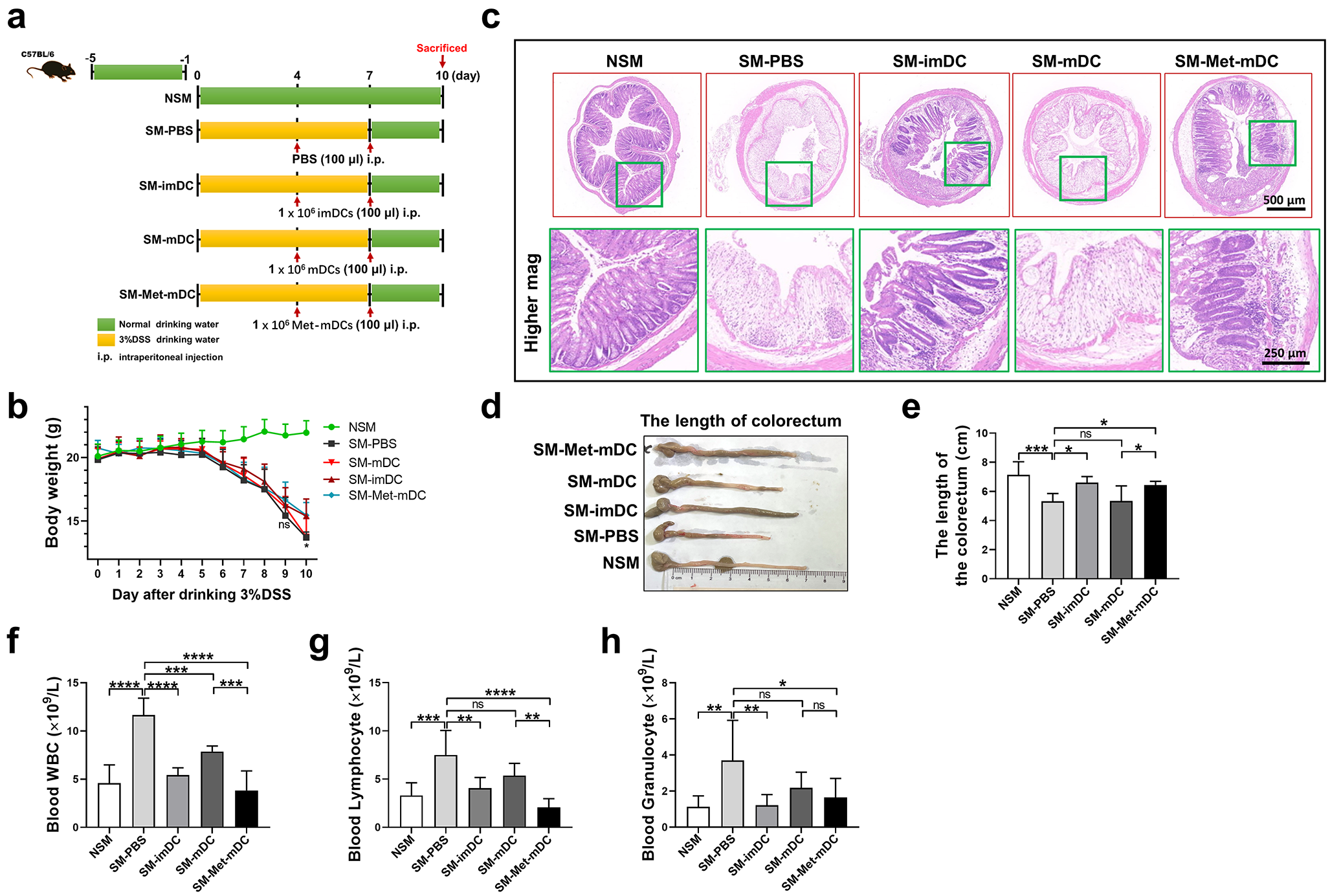

Supplement: Supplementary file 7 — Supplementary file7 (TIF 1687 KB) [file 18_2023_4932_MOESM7_ESM.tif]
