# Supplementary material for: Metformin induces tolerogenicity of dendritic cells by promoting metabolic reprogramming
Source: Cell Mol Life Sci. 2023 Sep 9;80(10):283. doi: 10.1007/s00018-023-04932-3 (PMC10492886; doi:10.1007/s00018-023-04932-3)
Supplement: Supplementary file 8 — Supplementary file8 (DOCX 19 KB) [file 18_2023_4932_MOESM8_ESM.docx]

**Supplemental figure captions:**

**Supplemental Fig. 1 Effects of metformin on the viability of DCs. (a)** Schematic of DC treatment with metformin. **(b)** The viabilities of imDCs treated with LPS and/or metformin at different concentrations for 24 or 48 h were detected by a CCK-8 kit. N=5. Paired Student’s t tests were used for comparisons between the 0 mM group and the other treatment groups. Mean ± SD *^*^p* < 0.05; *^**^p* < 0.01*.*

**Supplemental Fig. 2 Effects of metformin on the expression levels of immunophenotypic molecules in DCs.** **(a, b)** The expression levels of CD80, CD86, CD40, MHC-II, CCR7, HO-1, ICOSL, PD-L1 and PD-L2 were detected by RT‒qPCR. N=4-5. Paired Student’s t tests were used for comparisons between the 0 mM group/mDCs and other treatment groups/Met-mDCs. Mean ± SD *^*^p* < 0.05; *^**^p* < 0.01.

**Supplemental Fig. 3 KEGG pathway enrichment analysis of differential metabolites. (a)** Met-mDCs vs. imDCs. **(b)** imDCs vs. mDCs.

**Supplemental Fig. 4 Metabolites in imDCs, mDCs and Met-mDCs were detected by untargeted metabolic analysis. (a)** Related levels of FAs in imDCs, mDCs and Met-mDCs. **(b)** Related levels of citrate in imDCs, mDCs and Met-mDCs. **(c)** Related levels of the intermediates of the TAC in imDCs, mDCs and Met-mDCs. **(d)** Related levels of free amino acids in imDCs, mDCs and Met-mDCs. N=6. One-way ANOVA was used for comparisons among three groups. Mean ± SD **p* < 0.05; ***p* < 0.01; ****p* < 0.001.

**Supplemental Fig. 5 Mean fluorescence intensity (MFI) analysis of pFoxO3a in imDCs, mDCs and Met-mDCs.** N=6. One-way ANOVA was used for comparisons among three groups. Mean ± SD ***p* < 0.01; ****p* < 0.001.

**Supplemental Fig. 6 Metformin promoted the proportion of T_regs_ in the spleen and upregulated the expression level of PD-L1 in spleen-derived DCs from mice with IBD. (a, c)** Representative fluorescent images of CD4, Foxp3, CD11c, and PD-L1 in the spleen cells of mice in the DM and DP groups. From left to right are images of the four channels merged, nuclei (blue), CD4/CD11c (red), Foxp3/PD-L1 (green), double channels merged and partial enlarged images, respectively. **(b)** The proportion of T_regs_ in the spleen cells from mice in the NC, NP, DP and DM groups was measured by flow cytometry. N=3. **(d)** The expression level of PD-L1 in the splenic DCs of mice in the NC, NP, DP and DM groups was measured by flow cytometry. N=5. Unpaired Student’s t tests were used for two-group comparisons. Mean ± SD *^*^p* < 0.05; *^**^p* < 0.01.

**Supplemental Fig. 7 Metformin-modified DCs played anti-inflammatory roles and improved the clinical symptoms of mice with IBD. (a)** Diagram of model construction and the treatment of IBD. **(b)** Body weight changes in mice in the NSM, SM-PBS, SM-imDC, SM-mDC and SM-Met-mDC groups. N=6. **(c)** Representative H&E-stained colorectal sections of mice in the NSM, SM-PBS, SM-imDC, SM-mDC and SM-Met-mDC groups. N=6. **(d, e)** The length of the colorectum of mice in the NSM, SM-PBS, SM-imDC, SM-mDC and SM-Met-mDC groups. N=6. **(f, g, h)** The white blood cell (f), lymphocyte (g), and granulocyte (h) counts in the peripheral blood of mice in the NSM, SM-PBS, SM-imDC, SM-mDC and SM-Met-mDC groups. N=6. Unpaired Student’s t tests were used for weight comparisons between SM-mDC and SM-Met-mDC group. One-way ANOVA was used for multiple group comparisons. Mean ± SD *^*^p* < 0.05; *^**^p* < 0.01; *^***^p* < 0.001; *^****^p* < 0.0001.
