# Supplementary material for: Metformin induces tolerogenicity of dendritic cells by promoting metabolic reprogramming
Source: Cell Mol Life Sci. 2023 Sep 9;80(10):283. doi: 10.1007/s00018-023-04932-3 (PMC10492886; doi:10.1007/s00018-023-04932-3)
Supplement: Supplementary file 9 — Supplementary file9 (DOCX 15 KB) [file 18_2023_4932_MOESM9_ESM.docx]

**Supplemental tables:**

**Table 1. The list of primer sequences**

| Gene | Primer sequence |
| --- | --- |
| GAPDH | Forward 5’- ACCACAGTCCATGCCATCAC-3’  Reverse 5’- TCCACCACCCTGTTGCTGTA-3’ |
| CD11c | Forward 5’- GGATCTGGGATGCTGAAATC-3’  Reverse 5’- CAGTCTGGCAGATGTGGCTA-3’ |
| CD80 | Forward 5’- GGCAAGGCAGCAATACCTTA-3’  Reverse 5’- CCATGTCCAAGGCTCATTCT-3’ |
| CD86 | Forward 5’- GGCAGATATGCAGTCCCATT-3’  Reverse 5’- AGAACTTACGGAAGCACCCA-3’ |
| CD40 | Forward 5’- CTGGCACAAATCACAGCACT-3’  Reverse 5’- CTGCATGGTGTCTTTGCCT-3’ |
| MHC-II | Forward 5’- ACCGTGTTCTGCTCATCCT-3’  Reverse 5’- AGTTCTCAAAGTAGTGCCT-3’ |
| CCR7 | Forward 5’- GAAGGCTGTGCTTTTGGTTC-3’  Reverse 5’- CAAACAGGAGCTGATGTCCA-3’ |
| PD-L1 | Forward 5’- TGAGCAAGTGATTCAGTTTGTG-3’  Reverse 5’- CATTTCCCTTCAAAAGCTGGTC-3’ |
| PD-L2 | Forward 5’- CCCTAAAGAAGTGTACACCGTA-3’  Reverse 5’- CTTCTGCAAACTGGCTCTTATC-3’ |
| ICOSL | Forward 5’- GTTTGGAAGAAGCTCCATGTTT -3’  Reverse 5’- CAGGTAGTAAGTCACCGAAACT -3’ |
| HO-1 | Forward 5’- TCCTTGTACCATATCTACACGG -3’  Reverse 5’- GAGACGCTTTACATAGTGCTGT -3’ |
| Slc2a1 | Forward 5’- AGCAGAGGCTTGCTTGTAGAG -3’  Reverse 5’- GCCCGTCACCTTCTTGCT-3’ |
| Slc2a3 | Forward 5’- AATAGGTAGGCTGGGCTTCG-3’  Reverse 5’- AGATGGGGTCACCTTCGTT-3’ |
| Ldha | Forward 5’- AACTTGGCGCTCTACTTGCT-3’  Reverse 5’- GGACTTTGAATCTTTTGAGACCTTG-3’ |
| Acc1 | Forward 5’- TCCGTCGGTGGTCTTATGAA-3’  Reverse 5’- TGTTGTTGTTGGGTCCTCCA-3’ |
| Cpt1b | Forward 5’- AGGCACTTCTCAGCATGGTC-3’  Reverse 5’- ACGGACACAGATAGCCCAGA-3’ |

**Table 2.** **Grading rules of disease activity index**

| Disease activity index scoring | | | | | |
| --- | --- | --- | --- | --- | --- |
| Weight loss | Score | Hematochezia | Score | Stool consistency | Score |
| <1% | 0’ | Normal (hemoccult negative, no visible blood in stool) | 0’ | Normal (well-formed pellet, solid) | 0’ |
| 1%-5% | 1’ | Hemoccult positive (hemoccult positive, no visible blood in stool) | 1’ | Soft (well-formed pellet, soft) | 1’ |
| 5%-10% | 2’ | Slightly visible blood in stool (hemoccult positive, visible blood in stool with reddish hue upon smear) | 2’ | Pasty (semiformed pellet, readily becomes paste upon handling) | 2’ |
| 10%-15% | 3’ | Visible blood in stool (hemoccult positive, obvious blood in stool, but no incrustation around anus) | 3’ | Loose (poorly formed pellet, readily becomes paste upon  handling) | 3’ |
| >15% | 4’ | Gross bleeding (fresh extensive blood around anus or encrusted on fur) | 4’ | Diarrhea (no pellet formation, and/or liquid stools) | 4’ |
